# Effects of dapagliflozin and *n*-3 carboxylic acids on non-alcoholic fatty liver disease in people with type 2 diabetes: a double-blind randomised placebo-controlled study

**DOI:** 10.1007/s00125-018-4675-2

**Published:** 2018-07-03

**Authors:** Jan W. Eriksson, Per Lundkvist, Per-Anders Jansson, Lars Johansson, Mats Kvarnström, Linda Moris, Tasso Miliotis, Gun-Britt Forsberg, Ulf Risérus, Lars Lind, Jan Oscarsson

**Affiliations:** 1 Department of Medical Sciences, Uppsala University, Uppsala University Hospital, 751 85 Uppsala, Sweden; 2 Department of Molecular and Clinical Medicine, University of Gothenburg, Gothenburg, Sweden; 3 Antaros Medical AB, Gothenburg, Sweden; 4 AstraZeneca Gothenburg, Gothenburg, Sweden; 5 Karolinska Trial Alliance, Karolinska University Hospital, Stockholm, Sweden; 6 Department of Public Health and Caring Sciences, Uppsala University, Uppsala, Sweden

**Keywords:** Dapagliflozin, Docosahexaenoic acid, Eicosapentaenoic acid, Liver steatosis, Non-alcoholic fatty liver disease, Omega-3 fatty acids, Proton density fat fraction, Type 2 diabetes

## Abstract

**Aims/hypothesis:**

The EFFECT-II study aimed to investigate the effects of dapagliflozin and omega-3 (*n*-3) carboxylic acids (OM-3CA), individually or combined, on liver fat content in individuals with type 2 diabetes and non-alcoholic fatty liver disease (NAFLD).

**Methods:**

This randomised placebo-controlled double-blind parallel-group study was performed at five clinical research centres at university hospitals in Sweden. 84 participants with type 2 diabetes and NAFLD were randomly assigned 1:1:1:1 to four treatments by a centralised randomisation system, and all participants as well as investigators and staff involved in the study conduct and analyses were blinded to treatments. Each group received oral doses of one of the following: 10 mg dapagliflozin (*n* = 21), 4 g OM-3CA (*n* = 20), a combination of both (*n* = 22) or placebo (*n* = 21). The primary endpoint was liver fat content assessed by MRI (proton density fat fraction [PDFF]) and, in addition, total liver volume and markers of glucose and lipid metabolism as well as of hepatocyte injury and oxidative stress were assessed at baseline and after 12 weeks of treatment (completion of the trial).

**Results:**

Participants had a mean age of 65.5 years (SD 5.9), BMI 31.2 kg/m^2^ (3.5) and liver PDFF 18% (9.3). All active treatments significantly reduced liver PDFF from baseline, relative changes: OM-3CA, −15%; dapagliflozin, −13%; OM-3CA + dapagliflozin, −21%. Only the combination treatment reduced liver PDFF (*p* = 0.046) and total liver fat volume (relative change, −24%, *p =* 0.037) in comparison with placebo. There was an interaction between the *PNPLA3* I148M polymorphism and change in liver PDFF in the active treatment groups (*p =* 0.03). Dapagliflozin monotherapy, but not the combination with OM-3CA, reduced the levels of hepatocyte injury biomarkers, including alanine aminotransferase, aspartate aminotransferase, γ-glutamyl transferase (γ-GT), cytokeratin (CK) 18-M30 and CK 18-M65 and plasma fibroblast growth factor 21 (FGF21). Changes in γ-GT correlated with changes in liver PDFF (ρ *=* 0.53, *p =* 0.02). Dapagliflozin alone and in combination with OM-3CA improved glucose control and reduced body weight and abdominal fat volumes. Fatty acid oxidative stress biomarkers were not affected by treatments. There were no new or unexpected adverse events compared with previous studies with these treatments.

**Conclusions/interpretation:**

Combined treatment with dapagliflozin and OM-3CA significantly reduced liver fat content. Dapagliflozin monotherapy reduced all measured hepatocyte injury biomarkers and FGF21, suggesting a disease-modifying effect in NAFLD.

**Trial registration:**

ClinicalTrials.gov NCT02279407

**Funding:**

The study was funded by AstraZeneca.

**Electronic supplementary material:**

The online version of this article (10.1007/s00125-018-4675-2) contains peer-reviewed but unedited supplementary material, which is available to authorised users.

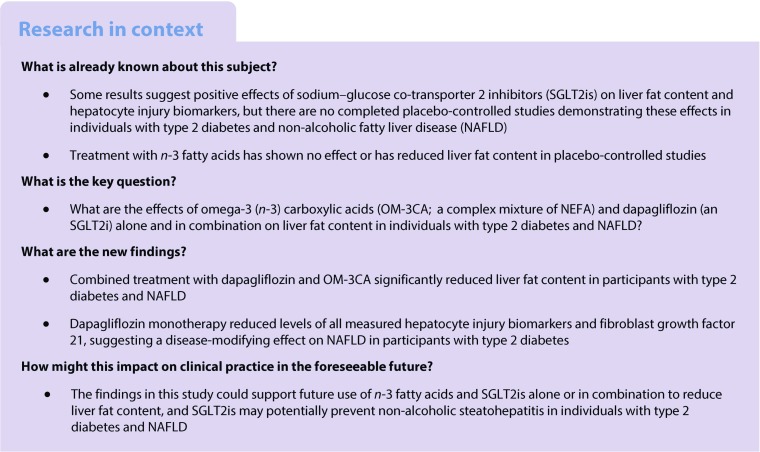



## Introduction

Non-alcoholic fatty liver disease (NAFLD) is defined as increased liver fat levels >5.5% and is associated with obesity, type 2 diabetes, chronic kidney disease and cardiovascular disease [1, 2]. The prevalence of NAFLD in individuals with type 2 diabetes is approximately 75% [1, 2]. Based on histological classification from liver biopsies, NAFLD can be classified as either non-alcoholic fatty liver (NAFL) or non-alcoholic steatohepatitis (NASH), which is the more aggressive form. Individuals with type 2 diabetes have a higher risk of developing NASH, and the disease has a more severe prognosis [1, 2]. The mechanisms responsible for progression from NAFL to NASH may involve lipotoxicity, oxidative stress, endoplasmic reticulum stress and mitochondrial dysfunction [3, 4]. Moreover, there are common genetic variants predisposing to both NAFL and NASH, including the well-documented I148M variant of the patatin-like phospholipase domain-containing protein 3 (*PNPLA3*) gene [5]. Currently, there are no approved drugs for treatment of NASH, and the recommended treatment consists of weight loss and exercise [2, 3].

Dapagliflozin is a sodium–glucose co-transporter 2 inhibitor (SGLT2i) indicated for the treatment of type 2 diabetes. SGLT2is increase urinary glucose excretion and reduce HbA_1c_, body weight, body fat and blood pressure [6, 7]. SGLT2is can also reduce the risk of cardiovascular events and death when added to standard of care [8–10]. To date, there are no completed placebo-controlled studies of the effects of SGLT2is on liver fat and hepatocyte injury biomarkers in individuals with type 2 diabetes and NAFLD, but some results suggest positive effects [11–13].

Omega-3 (*n*-3) carboxylic acids (OM-3CA) are a complex mixture of NEFA, a new chemical entity based on the premise that *n*-3 fatty acids in the free acid form have comparatively greater bioavailability than the prodrug ethyl ester or triacylglycerol forms [14]. A meta-analysis has shown that *n*-3 fatty acid treatment of NAFLD can lead to a reduction in liver fat content in the absence of weight loss [15]. In most studies, *n*-3 fatty acid supplementation had no significant effects on NASH histology [16–18], but high doses of *n*-3 fatty acids in combination with weight reduction improved liver histology compared with weight loss alone [19]. Overall, no effects of *n*-3 fatty acid treatment on glucose control in individuals with type 2 diabetes have been found [20]. Fatty acid oxidation and basal metabolic rate are increased by *n*-3 fatty acids, which could explain the reduced liver fat content without associated weight reduction [21]. In addition, *n*-3 fatty acid treatment has been shown to reduce total fat mass and adipocyte diameter [22], which is associated with reduced fatty acid release and inflammation, as well as improved insulin sensitivity [23].

The primary aim of this study was to evaluate the efficacy of treatment with a combination of OM-3CA and dapagliflozin, compared with placebo, on liver proton density fat fraction (PDFF) measured by MRI of the whole liver in individuals with type 2 diabetes and NAFLD. The secondary aim was to evaluate the relative efficacy of treatment with the combination of OM-3CA and dapagliflozin vs monotherapy with OM-3CA or dapagliflozin on reduction in liver PDFF. The effects of these treatments on glucose control, fatty acid metabolism, oxidative stress and hepatocyte injury biomarkers were also studied.

## Methods

### Study design

The EFFECT-II trial (ClinicalTrials.gov identifier NCT02279407) was a 12 week multicentre randomised placebo-controlled double-blind double-dummy four-armed parallel-group trial performed at five clinical research centres at university hospitals in Sweden. The first participant was enrolled on 20 January 2015, and the last patient visit was on 11 December 2015. The study was approved by the Regional Ethics Review Board in Uppsala, registered at ClinicalTrials.gov and conducted in accordance with the Declaration of Helsinki and the International Conference on Harmonisation of Good Clinical Practice. All participants provided written informed consent before participating.

### Participants

Individuals with type 2 diabetes, aged 40–75 years, were eligible if they had been treated with a stable dose of metformin or sulfonylurea alone or in combination for at least 3 months, and if they had a PDFF >5.5% (as measured by MRI), which is commonly used as a cut-off for NAFLD [2], and a BMI of 25–40 kg/m^2^. Exclusion criteria included the use of SGLT2is, *n*-3 fatty acids, insulin or glucagon-like peptide 1 receptor agonists, a history of hepatic disease, creatinine clearance <60 ml/min (Cockcroft–Gault), inability to undergo MRI scanning and a significant alcohol intake (>14 drinks per week). Complete inclusion and exclusion criteria are listed in the electronic supplementary material (ESM) Methods.

### Intervention

Participants who were eligible according to the inclusion and exclusion criteria were randomly assigned 1:1:1:1 to treatments by a centralised system, which provided a randomisation code delivered by an external call centre. They received either once-daily dapagliflozin 10 mg (Tablet Forxiga 10 mg, AstraZeneca, Södertälje, Sweden), OM-3CA 4 g (Capsule Epanova 1 g, AstraZeneca), a combination of dapagliflozin 10 mg and OM-3CA 4 g, or matching placebos. All participants as well as investigators and staff involved in the study conduct or analyses were blinded to treatments. The NEFA content of OM-3CA capsules is given in the ESM Methods. The study flow chart is shown in ESM Fig. 1. The randomisation schedule was stratified by baseline liver PDFF at two levels: ≤8% or > 8%.

### Outcome measures

#### Blood analyses

Detailed descriptions of examinations and analyses are available in the ESM Methods. Analyses were performed at baseline and after 12 weeks of treatment. In brief, glucose, NEFA, cholesterol, triacylglycerols, β-hydroxybutyrate and uric acid were determined with enzymatic colorimetric assays. HbA_1c_ was determined with ion-exchange high-performance liquid chromatography. Insulin, C-peptide, apolipoprotein C3, C-reactive protein, cytokeratin (CK) 18 fragments, adiponectin, fibroblast growth factor 21 (FGF21), leptin, TNF-α and osteopontin were analysed using immunoassays.

#### Glucose tolerance and insulin sensitivity indices

Plasma samples for measurement of glucose, NEFA and insulin were taken at −15, 0, 30, 60 and 120 min during a 75 g OGTT. Adipose tissue insulin sensitivity index was based on NEFA and insulin levels during the OGTT (ESM Methods).

#### Fatty acid composition of phospholipids and cholesteryl esters and total plasma levels of docosahexaenoic acid and eicosapentaenoic acid

The percentage composition of fatty acids in phospholipids and cholesteryl esters was determined by gas chromatography with flame ionisation detection. Desaturase activities were estimated by calculating fatty acid product to precursor ratios in plasma cholesteryl esters and phospholipids. Total eicosapentaenoic acid (EPA) and docosahexaenoic acid (DHA) (esterified and free) were measured by Covance Laboratories (Madison, WI, USA), on behalf of AstraZeneca, using LC-MS/MS (see ESM Methods).

#### *PNPLA3* genotyping

The single nucleotide polymorphism in the *PNPLA3* gene rs738409 was determined in *n =* 80 participants (*n =* 20 in each group) who gave informed consent for genetic testing. DNA was extracted from baseline samples of whole blood and genotypes were determined using quantitative real-time PCR (see ESM Methods).

#### MRI

MRI was used to quantify PDFF content, determined by the median of the fat fraction values inside the delineated total liver volume, and abdominal adipose tissue volumes (see ESM Methods for details).

#### Oxidative stress biomarkers in plasma and urine

Acylcarnitines, 4-hydroxyhexenal and 4-hydroxynonenal were analysed in plasma and non-esterified F2 isoprostanes were measured in urine samples using LC-MS. Creatinine was also measured in urine using an enzymatic colorimetric assay and used for normalisation of non-esterified F2 isoprostane levels (as described in ESM Methods).

### Statistical analysis

A sample size of 20 participants per treatment group was estimated to provide ≥90% power for the primary endpoint, accounting for a dropout rate of ≤10% and assuming a relative reduction of 30% liver PDFF for the combination of OM-3CA and dapagliflozin compared with placebo. The SDs used in the sample size estimates are based on the variations in change from baseline of PDFF on the natural log scale observed with in-house PDFF results, which are similar to the reported pooled SD of 0.24 (log scale) [24]. For the power calculation, a common SD of 0.17 (log scale) for the active treatment groups and 0.34 (log scale) for the placebo group were used. The study was also powered for the secondary objective involving three comparisons between the active treatment groups for liver PDFF. With 20 participants randomised per group, and under the same assumptions as above, the study had 80% power to reject the two null hypotheses of equal treatment effect of the combination of dapagliflozin and OM-3CA vs dapagliflozin alone and vs OM-3CA alone, assuming 15% relative difference.

Treatment effects were assessed using a mixed linear model with the change from baseline on a log scale as response variable, the logarithm of the baseline value as covariate, treatment as fixed effect and centre as random effect [log(POST) − log(BASE) ~ log(BASE) + TRT + centre (random)], where TRT is treatment. The resulting least-squares mean (LSM) treatment effects on a log scale were then back-transformed to the original scale. Herein, the results are described as the descriptive geometric mean ratio (GMR; back-transformed LSM estimates). The primary hypotheses were tested using three pairwise comparisons against placebo with respect to liver PDFF reduction using Dunnett’s multiple testing procedure. On conditional rejection of at least one of the primary hypotheses, all remaining pairwise comparisons between the active groups were carried out using Tukey’s method with a familywise error rate of 5%, adjusted for three pairwise comparisons. Results are presented as change (SD) from baseline as well as descriptive GMR with 95% CIs. Pairwise correlation analyses of changes from baseline to end of study were undertaken using the non-parametric Spearman’s rank correlation test. Interactions between baseline variables, including the effect of *PNPLA3* genotype, baseline PDFF and treatment on changes in liver PDFF in the active treatment groups, as well as the effect of baseline variables on changes in hepatocyte injury biomarkers in the dapagliflozin-treated group, were investigated using a mixed model on the log scale. Changes in liver PDFF or in hepatocyte injury biomarkers were used as responses. Baseline levels of these variables and others as specified were used as explanatory covariates and study site as a random effect covariate.

## Results

### Participants

Of 204 screened individuals, 114 were not eligible and six withdrew for other reasons. The remaining 84 participants were randomised (ESM Fig. 1) and constituted the safety and full analysis set (Table 1). At baseline, mean liver PDFF in all participants was 18%, HbA_1c_ 58 mmol/mol (7.4%), C-peptide level 0.98 nmol/l and fasting plasma glucose 9.4 mmol/l. Fewer than 10% of participants reported diabetes-related complications. Most individuals were treated with metformin or a sulfonylurea (82% and 18%, respectively) alone or in combination, and 14% were drug naive. No change in this medication occurred during the study. Participants were randomised to four groups: placebo (*n =* 21), OM-3CA monotherapy (*n =* 20), dapagliflozin monotherapy (*n =* 21) and combined OM-3CA and dapagliflozin therapy (*n =* 22). Compliance was high (97%) and similar in each treatment group. In total, 75 participants (89%) completed the study (ESM Fig. 1).

**Table 1:** Participant characteristics at baseline

Characteristic	Placebo (*n =* 21)	OM-3CA (*n =* 20)	Dapagliflozin (*n =* 21)	OM-3CA + dapagliflozin (*n =* 22)	Total (*N =* 84)
Age, years	65.6 (6.1)	66.2 (5.9)	65.0 (6.5)	65.0 (5.4)	65.5 (5.9)
Sex, male/female, *n*/*n*	17/4	11/9	16/5	15/7	59/25
Weight, kg	93.0 (12.2)	95.6 (13.7)	90.2 (8.7)	91.7 (12.9)	92.6 (12.0)
BMI, kg/m^2^	30.3 (3.1)	33.0 (4.1)	30.5 (2.8)	31.1 (3.6)	31.2 (3.5)
Overweight/obese^a^, *n*/*n*	10/11	5/15	11/10	10/12	36/48
Diabetes duration, years	6.5 (4.2)	6.3 (5.1)	6.7 (6.0)	8.5 (4.5)	7.0 (5.0)

Data are reported as mean (SD), unless otherwise stated

^a^Overweight, BMI 25–30 kg/m^2^; obese, BMI > 30 kg/m^2^

### Liver fat content

**Fig. 1:**
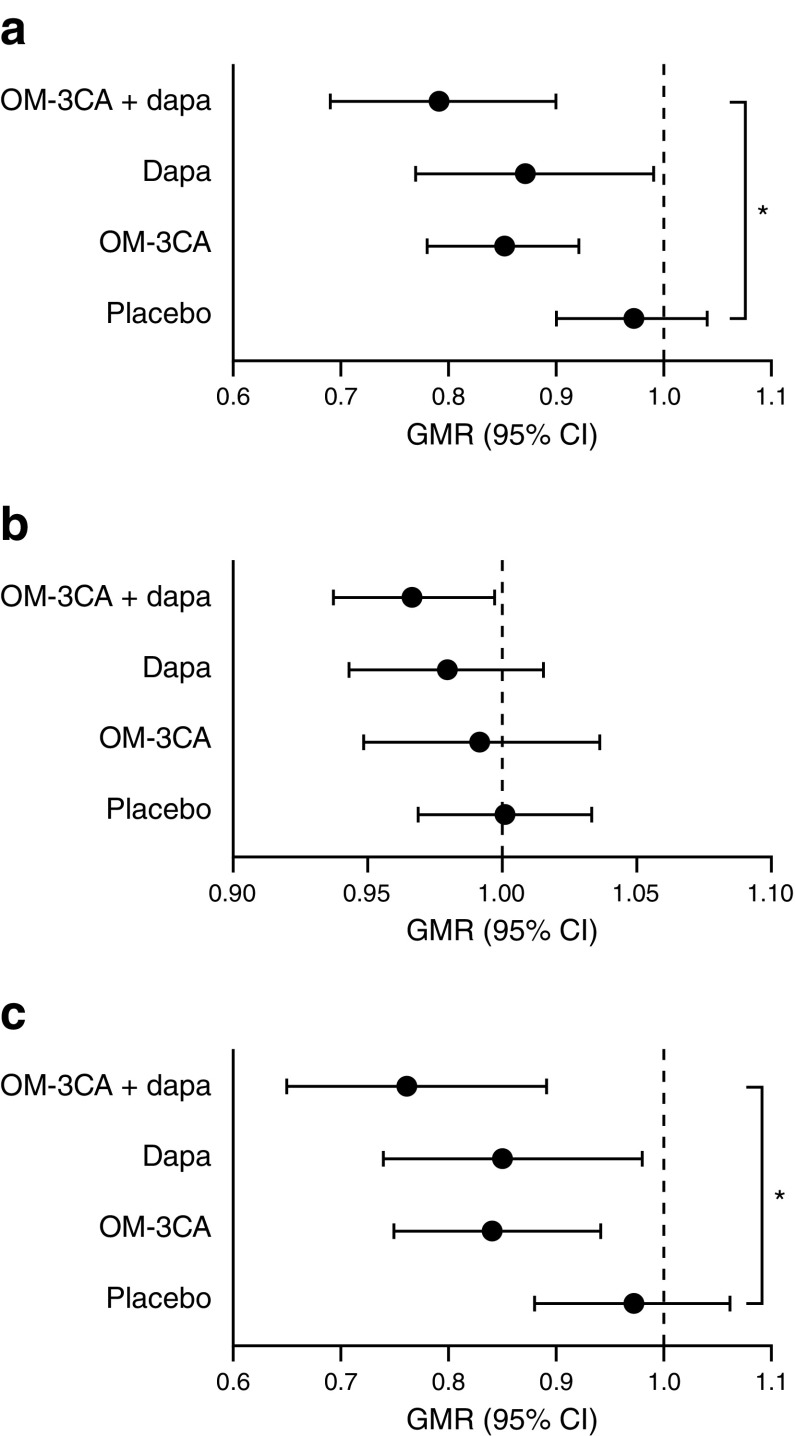
Effects of dapagliflozin and OM-3CA on liver PDFF (%) (**a**), liver volume (l) (**b**) and total liver fat (l) (**c**). Data are descriptive GMRs of post-treatment to baseline values with 95% CIs. ^*^p < 0.05 vs placebo. Dapa, dapagliflozin

Dapagliflozin and OM-3CA, on their own or in combination, significantly reduced liver PDFF from baseline to 12 weeks (Table 2 and Fig. 1). The combination treatment (relative change, −21%, adjusted *p* < 0.05), but not dapagliflozin (−13%) or OM-3CA (−15%) as monotherapies, significantly lowered liver PDFF compared with placebo (−3%). Total liver fat volume calculated from total liver volume and PDFF changed similarly to liver PDFF (Table 2 and Fig. 1), suggesting that the change in PDFF was not secondary to changes in hepatic fat-free volume.

**Table 2:** Treatment effects on body weight, waist and hip circumference, abdominal adipose tissue, and liver fat and volume

Variable	Placebo (*n =* 19)	OM-3CA (*n =* 15)	Dapagliflozin (*n =* 19)	OM-3CA + dapagliflozin (*n =* 20)
Body weight, kg				
Baseline	92.9 (12.16)	95.6 (13.68)	90.3 (9.04)	91.6 (12.84)
Change	−0.27 (1.79)	−0.16 (1.02)	−2.44 (2.14)	−2.16 (1.30)
GMR	1.00 (0.99, 0.01)	1.00 (0.99, 1.00)	0.97 (0.96, 0.98)^*^	0.98 (0.97, 0.98)^*^
Waist circumference, cm				
Baseline	109.9 (7.4)	114.5 (9.7)	110.1 (8.5)	110.7 (8.7)
Change	0.9 (3.6)	0.1 (2.2)	−2.2 (3.3)	−2.2 (3.2)
GMR	1.01 (0.99, 1.02)	1.00 (0.99, 1.01)	0.98 (0.97, 1.00)^*^	0.98 (0.97, 0.99)^*^
Hip circumference, cm				
Baseline	107.5 (7.8)	112.6 (9.4)	106.2 (6.8)	107.5 (7.3)
Change	0.3 (4.4)	−0.6 (3.8)	−2.3 (4.2)	−0.4 (4.2)
GMR	1.00 (0.98, 1.02)	0.99 (0.97, 1.01)	0.98 (0.96, 1.00)^*^	1.00 (0.98, 1.01)
Liver PDFF, %				
Baseline	15.1 (6.5)	22.2 (11.0)	17.3 (9.1)	17.8 (9.2)
Change	−0.59 (1.86)	−3.15 (2.88)	−2.23 (3.30)	−3.15 (3.49)
GMR	0.97 (0.90, 1.04)	0.85 (0.78, 0.92)	0.87 (0.77, 0.99)	0.79 (0.69, 0.90)^*^
Total liver volume, l				
Baseline	1.91 (0.35)	2.30 (0.70)	1.88 (0.44)	1.88 (0.51)
Change	−0.001 (0.120)	−0.025 (0.165)	−0.049 (0.153)	−0.060 (0.129)
GMR	1.00 (0.97, 1.03)	0.99 (0.95, 1.04)	0.98 (0.94, 1.01)	0.97 (0.94, 1.00)
Total liver fat volume, l				
Baseline	0.30 (0.17)	0.51 (0.37)	0.37 (0.28)	0.36 (0.25)
Change	−0.01 (0.04)	−0.07 (0.11)	−0.06 (0.09)	−0.07 (0.08)
GMR	0.97 (0.88, 1.06)	0.84 (0.75, 0.94)	0.85 (0.74, 0.98)	0.76 (0.65, 0.89)^*^
Subcutaneous adipose tissue volume, l				
Baseline	3.91 (1.59)	4.93 (2.03)	3.84 (1.40)	4.10 (1.53)
Change	−0.06 (0.22)	0.05 (0.13)	−0.29 (0.28)	−0.23 (0.22)
GMR	0.98 (0.95, 1.01)	1.01 (0.99, 1.04)	0.92 (0.90, 0.95)^*^	0.94 (0.91, 0.96)^*^
Visceral adipose tissue volume, l				
Baseline	3.96 (1.06)	4.32 (1.20)	4.02 (1.03)	3.95 (1.02)
Change	0.03 (0.32)	0.01 (0.20)	−0.27 (0.25)	−0.17 (0.23)
GMR	1.01 (0.97, 1.05)	1.00 (0.98, 1.03)	0.93 (0.90, 0.96)^*^	0.96 (0.93, 0.99)^*^

Baseline and change are reported as mean (SD)

Descriptive GMR reported with 95% CIs

Change is change from baseline to end of treatment

^*^p < 0.05 vs placebo, mixed model analysis

The baseline NAFLD fibrosis score was calculated [25]. Eight participants (*n =* 2 in OM-3CA group, *n =* 4 in the dapagliflozin group and *n =* 2 in the combination group) had an NAFLD fibrosis score >0.675, indicating significant fibrosis. The NAFLD fibrosis score did not interact significantly with the effect of treatment on liver PDFF.

The I148M genetic variant of *PNPLA3* (rs738409 C>G) has been reported to increase liver fat content and risk of developing NASH [5]. Therefore, the participants with the most common genotype C/C (*n =* 47) were compared with those with the C/G (*n =* 30) and G/G genotypes (*n =* 3). The proportions of the C/C and C/G + G/G genotypes did not differ significantly between the treatment groups. Baseline liver PDFF was numerically lower in the C/C group (median [range]: 17.4% [8.3–34.7%]) than in the C/G + G/G group (20.0% [6.1–48.5%]; *p =* 0.19). There was a significant interaction between *PNPLA3* genotype (C/C vs C/G + G/G) and treatment response on liver PDFF across the active treatment arms (*p =* 0.03). In the combination treatment group, the relative reduction in liver PDFF was numerically larger for the C/G + G/G genotype (relative change: −25.4% [−27.3 to −19.0%]) than for the C/C genotype (−16.1% [−20.5 to −11.6%]), and this was significantly different (*p* < 0.01) from that seen with dapagliflozin alone (C/G + G/G: 7.0% [−2.2 to 11.3%]; C/C: −22.0% [−26.8 to −19.2%]; *p* < 0.01). In the OM-3CA treatment group, the change in liver PDFF was numerically smaller for the C/G + G/G genotype (−12.6% [−15.9 to −4.3%]) than for the C/C genotype (−18.6% [−20.2 to −15.6%]), but this was not significantly different from other treatment groups.

### Anthropometrics and abdominal adipose tissue volumes

Participants using dapagliflozin alone and in combination with OM-3CA showed reduced body weight and waist circumference, whereas those using OM-3CA alone showed no change compared with the placebo group (Table 2). Abdominal subcutaneous and visceral adipose tissue volumes decreased in the two groups treated with dapagliflozin. There was a significant interaction between the baseline subcutaneous fat volume and treatment response in the active treatment arms on liver PDFF (*p =* 0.017), which reached statistical significance in the group treated with both dapagliflozin and OM-3CA vs the group treated with dapagliflozin alone (*p =* 0.006).

### Glucose metabolism

In participants using dapagliflozin, HbA_1c_ decreased from baseline, but this effect was significant only in individuals using dapagliflozin monotherapy when compared with the placebo group (Table 3). In participants using dapagliflozin, fasting and 2 h plasma glucose concentrations decreased, while OM-3CA treatment had no effect (Table 3). Fasting insulin levels were reduced from baseline in all participants using dapagliflozin, but the effect was not significant vs the placebo group. Dapagliflozin treatment significantly improved the insulin sensitivity index measured using HOMA-IR, while OM-3CA treatment had no effect. Plasma levels of NEFA and the insulin sensitivity index for NEFA/lipolysis [26] were not affected by any treatment. The changes in 2 h plasma glucose and insulin levels in the dapagliflozin monotherapy group correlated with the changes found in liver PDFF (ρ *=* 0.55, *p =* 0.02 and ρ *=* 0.62, *p =* 0.005, respectively; ESM Fig. 2).

**Table 3:** Treatment effects on glucose, HbA_1c_, insulin, HOMA-IR, NEFA and insulin sensitivity index for lipolysis

Variable	Placebo (*n =* 20)	OM-3CA (*n =* 14)	Dapagliflozin (*n =* 20)	OM-3CA + dapagliflozin (*n =* 19)
HbA_1c_^a^				
Baseline, mmol/mol	57.9 (9.0)	57.3 (8.4)	56.7 (6.1)	58.9 (8.9)
Baseline, %	7.44 (0.80)	7.38 (0.68)	7.38 (0.56)	7.50 (0.76)
Change, mmol/mol	−0.99 (3.81)	1.43 (4.35)	−6.89 (7.24)	−4.88 (5.25)
Change, % units	−0.09 (0.35)	0.13 (0.40)	−0.63 (0.66)	−0.45 (0.48)
GMR	0.99 (0.97, 1.01)	1.01 (0.98, 1.04)	0.91 (0.87, 0.96)^*^	0.94 (0.92, 0.97)
Fasting glucose, mmol/l				
Baseline	9.40 (1.65)	9.02 (1.48)	8.99 (1.85)	9.38 (1.97)
Change	0.37 (0.82)	0.21 (1.07)	−0.98 (1.49)	−0.91 (2.00)
GMR	1.04 (0.99, 1.08)	1.02 (0.95, 1.09)	0.90 (0.84, 0.96)^*^	0.91 (0.83, 1.01)^*^
Fasting insulin, pmol/l				
Baseline	72.5 (40.0)	94.9 (39.2)	79.1 (38.5)	75.2 (28.3)
Change	−3.8 (18.3)	−4.0 (15.8)	−9.6 (24.6)	−10.7 (22.6)
GMR	0.94 (0.82, 1.07)	0.96 (0.84, 1.09)	0.84 (0.72, 0.98)	0.81 (0.71, 0.93)
HOMA-IR^b^				
Baseline	4.2 (2.4)	5.4 (2.9)	4.3(1.9)	4.4 (1.7)
Change	−0.19 (1.44)	0.31 (2.39)	−1.08 (1.38)	−0.86 (1.58)
GMR	0.97 (0.84, 1.12)	1.03 (0.83, 1.28)	0.72 (0.60, 0.86)^*^	0.76 (0.65, 0.90)^*^
120 min glucose^c^, mmol/l				
Baseline	17.5 (3.8)	16.9 (2.2)	17.2 (4.1)	16.8 (3.8)
Change	0.7 (1.9)	0.4 (0.8)	−2.2 (3.1)	−1.2 (2.2)
GMR	1.06 (0.99, 1.12)	1.02 (0.99, 1.06)	0.88 (0.78, 0.98)^*^	0.94 (0.88, 1.01)^*^
NEFA, mmol/l				
Baseline	0.76 (0.34)	0.64 (0.16)	0.64 (0.20)	0.66 (0.18)
Change	−0.08 (0.18)	0.02 (0.14)	0.04 (0.18)	0.02 (0.17)
GMR	0.91 (0.80, 1.03)	1.00 (0.84, 1.20)	1.07 (0.92, 1.23)	1.03 (0.90, 1.18)
Insulin sensitivity index for lipolysis^d^				
Baseline	0.79 (0.261)	0.64 (0.238)	0.79 (0.260)	0.79 (0.251)
Change	0.027 (0.123)	0.010 (0.171)	−0.022 (0.124)	0.027 (0.149)
GMR	1.04 (0.96, 1.13)	1.04 (0.90, 1.19)	0.99 (0.90, 1.08)	1.05 (0.94, 1.16)

Baseline and change are reported as mean (SD)

Descriptive GMR reported with 95% CIs

Change is change from baseline to end of treatment

^a^HbA_1c_: mmol/mol, International Federation of Clinical Chemistry (IFCC); %, National Glycohemoglobin Standardization Program (NGSP) units

^b^HOMA-IR is calculated as: glucose, mmol/l × insulin, pmol/l)/156

^c^Glucose levels at 120 min during the OGTT, as a measure of glucose tolerance

^d^Data are derived from measurements of insulin and NEFA at 0, 60 and 120 min during the OGTT

^*^p < 0.05 vs placebo, mixed model analysis

### Plasma fatty acid composition and lipoprotein levels

Concentrations of the fatty acids DHA and EPA were measured in total plasma and in the cholesteryl ester and phospholipid fractions (Table 4 and ESM Tables 1–4). DHA levels increased by 20–40%, while EPA levels increased about threefold in the different lipid fractions in participants using OM-3CA. Baseline levels of DHA or EPA did not interact significantly with the effect of treatment on liver PDFF, and there were no significant correlations between changes in DHA or EPA and changes in liver PDFF.

**Table 4:** Estimates of δ-5, δ-6 and δ-9 desaturase activities from fatty acid composition of cholesteryl esters

Variable	Placebo (*n =* 20)	OM-3CA (*n =* 16)	Dapagliflozin (*n =* 19)	OM-3CA + dapagliflozin (*n =* 20)
20:4 *n*-6/20:3 *n*-6 (δ-5 desaturase)				
Baseline	9.87 (2.42)	9.14 (2.37)	8.76 (1.98)	10.91 (1.97)
Change	0.26 (0.82)	3.53 (2.57)	1.24 (1.50)	3.79 (3.10)
GMR	1.02 (0.98, 1.06)	1.38 (1.22, 1.55)^*^	1.14 (1.07, 1.21)	1.33 (1.20, 1.47)^*^
18:3 *n*-6/18:2 *n*-6 (δ-6 desaturase)				
Baseline	0.022 (0.01)	0.025 (0.008)	0.021 (0.009)	0.023 (0.009)
Change	−0.002 (0.006)	−0.009 (0.006)	−0.001 (0.009)	−0.008 (0.006)
GMR	0.94 (0.85, 1.04)	0.66 (0.58, 0.76)^*^	0.95 (0.79, 1.15)	0.65 (0.56, 0.74)^*^
16:1 *n*-7/16:0 (δ-9 desaturase)				
Baseline	0.301 (0.112)	0.343 (0.089)	0.314 (0.131)	0.279 (0.080)
Change	−0.016 (0.047)	−0.070 (0.052)	−0.027 (0.086)	−0.072 (0.053)
GMR	0.96 (0.88, 1.03)	0.78 (0.72, 0.85)^*^	0.93 (0.82, 1.05)	0.74 (0.68, 0.80)^*^
18:1 *n*-9/18:0 (δ-9 desaturase)				
Baseline	30.7 (7.39)	29.2 (4.44)	30.7 (7.48)	32.0 (5.99)
Change	1.21 (5.34)	−1.29 (6.27)	1.97 (7.13)	−3.79 (7.34)
GMR	1.04 (0.96, 1.14)	0.95 (0.84, 1.08)	1.09 (0.96, 1.23)	0.88 (0.78, 1.00)

Baseline and change are reported as mean (SD)

Descriptive GMR reported with 95% CIs

Change is change from baseline to end of treatment

^*^p < 0.05 vs placebo, mixed model analysis

Changes in fatty acid composition in the cholesteryl ester and phospholipid fractions are shown in ESM Tables 2 and 3. OM-3CA treatment resulted in small and inconsistent changes in saturated fatty acids in the cholesteryl ester and phospholipid fractions. Levels of monounsaturated fatty acids (16:1 *n*-7, 18:1 *n*-9) and several *n*-6 fatty acids (18:2, 18:3, 20:3) decreased after OM-3CA treatment, while 18:3 *n*-3 and 20:4 *n*-6 levels did not change. Dapagliflozin treatment had no or inconsistent effects on fatty acid composition in the cholesteryl ester and phospholipid fractions (ESM Tables 2 and 3).

OM-3CA treatment increased estimated δ-5 desaturase activity and decreased δ-6 desaturase and stearoyl-CoA desaturase-1 (SCD-1) activity indices, while dapagliflozin treatment had no significant effect on these activities (Table 4 and ESM Table 4). The change in liver PDFF was significantly associated with change in SCD-1 index in the dapagliflozin group (ρ *=* 0.54, *p =* 0.02; ESM Fig. 2), but not in the other groups. Elongase activity index (cholesteryl ester 18:0 cholesteryl ester 16:0) was not significantly influenced by treatment (data not shown). Total, LDL- and HDL-cholesterol as well as triacylglycerol levels were not significantly changed by any treatment vs placebo (ESM Table 5). Apolipoprotein C3 levels increased following treatment with dapagliflozin, while no effect was seen in the OM-3CA groups.

Both dapagliflozin groups had increased β-hydroxybutyrate levels numerically, but not significantly vs the placebo group (ESM Table 5). No significant correlation between changes in β-hydroxybutyrate and liver PDFF was observed. Dapagliflozin treatment increased butyrylcarnitine levels, while there was no effect of the combination treatment on plasma levels of the acylcarnitines vs placebo (ESM Table 5).

### Hepatocyte injury, oxidative stress and inflammation biomarkers and adipokines

**Fig. 2:**
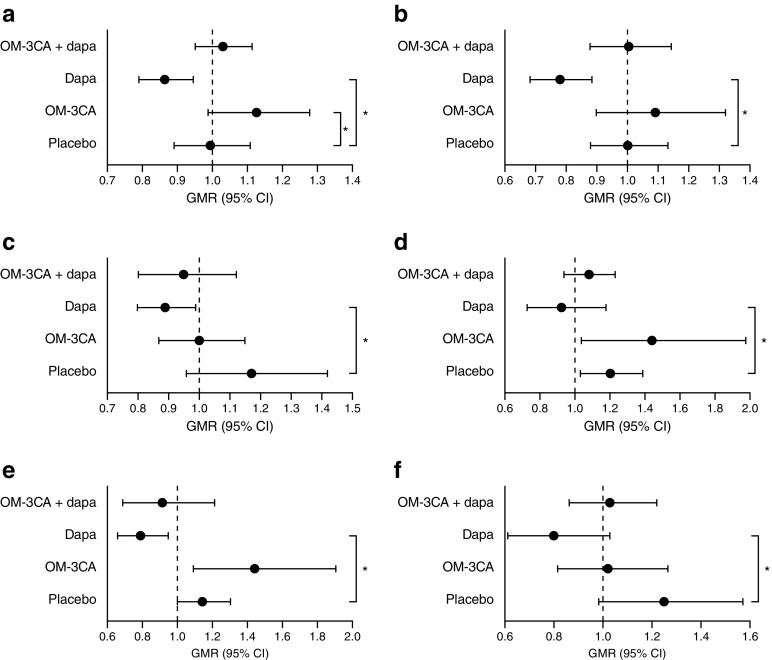
Effects of dapagliflozin and OM-3CA on levels of aspartate aminotransferase (μkat/l) (**a**), alanine aminotransferase (μkat/l) (**b**), γ-GT (μkat/l) (**c**), CK 18-M30 (U/l) (**d**), CK 18-M65 (U/l) (**e**) and plasma FGF21 (pg/ml) (**f**). Data are descriptive GMRs of post-treatment to baseline values with 95% CIs. ^*^p < 0.05 vs placebo. Dapa, dapagliflozin

Dapagliflozin monotherapy reduced levels of all measured hepatocyte injury biomarkers, including aspartate aminotransferase, alanine aminotransferase, γ-glutamyl transferase (γ-GT), CK 18-M30 and CK 18-M65 (Fig. 2, ESM Table 6). There was no significant effect of the OM-3CA monotherapy or the combination therapy on any of the hepatocyte injury biomarkers. Changes in liver PDFF correlated significantly with changes in γ-GT (ρ *=* 0.53, *p =* 0.02), but not with the other hepatocyte injury biomarkers in the dapagliflozin group. Uric acid levels were significantly reduced in the dapagliflozin and combination groups, but not with OM-3CA, compared with placebo (ESM Table 6).

Oxidative stress biomarkers related to non-enzymatic oxidation of unsaturated fatty acids were measured in plasma and urine (ESM Table 6). The *n*-3 fatty acid-derived oxidative stress biomarker 2-hydroxyhexenal was significantly increased by the OM-3CA and combination treatments. Levels of 8-iso-prostaglandin F2-α (8-iso-PGF2-α) were numerically lower in the OM-3CA group.

Plasma osteopontin levels increased significantly following OM-3CA + dapagliflozin, but not monotherapies, vs placebo. OM-3CA + dapagliflozin dual therapy had no significant effects on C-reactive protein, adiponectin and leptin levels (ESM Table 7). FGF21 levels decreased significantly in the dapagliflozin compared with the placebo group, but OM-3CA or combination treatments had no significant effect (ESM Table 7 and ESM Fig. 2). There was no significant association between changes in FGF21 and changes in liver PDFF, acylcarnitines or β-hydroxybutyrate in active treatment groups.

### Adverse events and safety

All active treatment groups had similar total percentages of adverse event reporting (70.0–77.3%), which were higher than in the placebo group (47.6%). There were no new or unexpected adverse events compared with previous studies with these treatments. More participants reported adverse events when using dapagliflozin and OM-3CA (*n =* 15, 68.2%) than when using dapagliflozin monotherapy (*n =* 7, 33.3%), OM-3CA monotherapy (*n =* 8, 40%) or placebo (*n =* 6, 28.6%). All adverse events were mild or moderate in intensity, except two serious adverse events judged by investigators as unlikely to be caused by study treatments. There were no significant changes in serum creatinine levels in any of the treatment groups.

## Discussion

This randomised controlled trial provides evidence for the effect of SGLT2is and *n*-3 fatty acids on liver fat content in individuals with type 2 diabetes and NAFLD measured as PDFF derived from the whole liver volume. The combined 12 week treatment of OM-3CA and the SGLT2i dapagliflozin significantly reduced liver fat content in individuals with type 2 diabetes and NAFLD. Thus, the study met its primary objective and provides proof of concept for this dual therapy to reduce liver fat in individuals with type 2 diabetes and NAFLD. Additionally, each of the treatments as monotherapy led to a significant liver fat reduction from baseline.

Interestingly, dapagliflozin alone reduced all hepatocyte injury biomarkers as well as plasma levels of FGF21, suggesting reduced cell damage and improved mitochondrial function or reduced endoplasmic reticulum stress in the liver [4, 27]. However, these effects of dapagliflozin were not seen when combined with OM-3CA, indicating a complex relationship between the two drugs and their effects on liver metabolism.

Dapagliflozin treatment was followed by the expected reductions in HbA_1c_, body weight, abdominal adipose volumes and uric acid. These are well-established effects that are in accordance with the mechanism of action [6, 7]. As expected, OM-3CA had no effects on these measures [20, 21].

In this study, we measured the PDFF from the entire liver volume excluding bile ducts and veins by semi-automated segmentation, instead of the common region-of-interest (ROI)-based analysis that typically uses 3–9 ROIs. Measurement of PDFF from the entire liver volume is a novel method expected to result in more accurate results and reduced variability. An effect of dapagliflozin on liver fat content may be explained by a negative energy balance via energy loss in the urine together with an increase in fatty acid oxidation [28] that might be promoted by an increased glucagon/insulin ratio [29]. Combining dapagliflozin with OM-3CA resulted in a numerically larger reduction in PDFF, which may be dependent on increased fatty acid oxidation and reduced fatty acid synthesis, effects of OM-3CA that are independent of weight reduction [21, 30]. We did not observe any change in adipose insulin sensitivity index, suggesting no change in fatty acid flux to the liver.

We found a significant interaction between the *PNPLA3* I148M polymorphism and treatment effects on liver PDFF. The influence of the C/C vs C/G + G/G genotypes appeared to differ between the treatment groups, in particular dapagliflozin alone vs combination therapy. Our results suggested that G allele carriers had a greater treatment response only in the combined OM3-CA and dapagliflozin group. In participants with the metabolic syndrome and NAFLD, the G/G genotype was previously reported to be associated with the largest reduction of liver fat following lifestyle intervention [31], whereas the response to treatment with *n*-3 fatty acids was lowest with this genotype [32]. Thus, future studies on effects of SGLT2is and *n*-3 fatty acids on NAFLD and NASH should include assessment of *PNPLA3* genetics.

Notably, dapagliflozin monotherapy led to reduced signs of hepatocellular injury as indicated by several biochemical markers. It is unclear why the addition of OM-3CA prevented this effect of dapagliflozin. One possibility is that OM-3CA increased the transcription of transaminases via peroxisome proliferator-activated receptor α as previously shown [33]. This, however, would not explain why OM-3CA prevented the dapagliflozin-induced reductions in γ-GT, CK 18-M30 and CK 18-M65 levels. Treatment with dapagliflozin reduced plasma levels of FGF21, while the combination with OM-3CA did not. High FGF21 levels are associated with NASH and mitochondrial dysfunction [4, 27, 34, 35]. It is therefore possible that reduced levels of FGF21 and hepatocyte injury biomarkers following dapagliflozin treatment reflect reduced metabolic stress and that OM-3CA treatment opposed these effects.

A significant percentage of the participants in this study are likely to have undiagnosed NASH based on their risk profile [2], and eight participants had signs of severe fibrosis indicated by their NAFLD fibrosis score. Reduced hepatocyte injury biomarkers suggest NASH resolution, as found in previous intervention studies such as the FLINT study [36]. The consistent reduction in hepatocyte injury biomarkers therefore suggests that dapagliflozin has a beneficial effect on NASH, which may be mediated via reduced lipotoxicity and oxidative stress, as supported by the lower liver fat content associated with reduced γ-GT levels.

Overall, there were no obvious effects of dapagliflozin on plasma fatty acid composition. However, changes in liver PDFF in the dapagliflozin group were correlated with changes in the SCD-1 index (16:1 *n*-7/16:0), which mostly reflects hepatic fatty acid metabolism [24] and is associated with liver fat content [24], NAFLD [37] and lobular inflammation [38]. Therefore, the association between changes in SCD-1 index and liver PDFF suggests that dapagliflozin may influence SCD-1 activity. Interestingly, OM-3CA decreased SCD-1 and δ-6 desaturase indices, but increased δ-5 desaturase index, an apparent enzyme activity pattern that has been associated with reduced risk of developing type 2 diabetes [39]. Levels of the major *n*-6 polyunsaturated fatty acids were decreased after OM-3CA treatments, probably reflecting substrate competition for desaturases among *n*-6 and *n*-3 fatty acids [39].

In a situation of incomplete fatty acid oxidation, acyl groups are exported from the mitochondria as acylcarnitines, which can be found in the circulation [40, 41]. Notably, butyrylcarnitine levels increased in the dapagliflozin treatment group. The mechanisms are unclear but may reflect an increased mitochondrial flux of 4-carbon molecules and fatty acids, as reflected by increased β-hydroxybutyrate levels [29].

The primary objective was to compare the effect of the combination of dapagliflozin and OM-3CA with that of placebo on liver PDFF, and the study was powered accordingly. However, the sample size was not sufficient to evaluate each monotherapy optimally vs other treatments.

Taken together, the results suggest that dapagliflozin or OM-3CA alone or in combination reduce liver fat content in overweight individuals with type 2 diabetes and NAFLD. In addition, the novel effects of dapagliflozin on hepatocyte injury biomarkers are promising for the prevention and treatment of NASH, but they should be considered as hypothesis-generating findings. Future studies would ideally include histological examination of repeated liver biopsies in individuals with NASH, as well as long-term follow-up on liver outcomes.

## Electronic supplementary material

ESM (PDF 853 kb)

## Data Availability

Data and the full study protocol are available upon request to the authors.

## Abbreviations

CK: Cytokeratin
DHA: Docosahexaenoic acid
EPA: Eicosapentaenoic acid
FGF21: Fibroblast growth factor 21
GMR: Geometric mean ratio
GT: Glutamyl transferase
LSM: Least-squares mean
NAFL: Non-alcoholic fatty liver
NAFLD: Non-alcoholic fatty liver disease
NASH: Non-alcoholic steatohepatitis
OM-3CA: Omega-3 (*n*-3) carboxylic acids
PDFF: Proton density fat fraction
ROI: Region-of-interest
SCD-1: Stearoyl-CoA desaturase 1
SGLT2i: Sodium–glucose co-transporter 2 inhibitor
