# Supplementary material for: Effects of dapagliflozin and n-3 carboxylic acids on non-alcoholic fatty liver disease in people with type 2 diabetes: a double-blind randomised placebo-controlled study
Source: Diabetologia. 2018 Jul 3;61(9):1923–34. doi: 10.1007/s00125-018-4675-2 (PMC6096619; doi:10.1007/s00125-018-4675-2)
Supplement: Supplementary file 1 — (PDF 853 kb) [file 125_2018_4675_MOESM1_ESM.pdf]

ESM final version, June 2018

Electronic supplementary materials

Effects of dapagliflozin and omega-3 carboxylic acids on non-alcoholic fatty liver disease in people with type 2 diabetes: a double-blind randomised placebo-controlled study

Jan W. Eriksson[1] • Per Lundkvist[1] • Per-Anders Jansson[2] • Lars Johansson[3] • Mats Kvarnström[4] • Linda Moris[5] • Tasso Miliotis[4] • Gun-Britt Forsberg[4] • Ulf Risérus[6] • Lars Lind[6] • Jan Oscarsson[4]

[1]Department of Medical Sciences, Uppsala University, Sjukhusvägen 85, 751 85 Uppsala, Sweden

[2]Department of Molecular and Clinical Medicine, University of Gothenburg, Gothenburg, Sweden

[3]Antaros Medical AB, Gothenburg, Sweden

[4]AstraZeneca R&D, Gothenburg, Sweden

[5]Karolinska Trial Alliance, Karolinska University Hospital, Stockholm, Sweden

[6]Department of Public Health and Caring Sciences, Uppsala University, Uppsala, Sweden

Corresponding author

Jan Eriksson

Department of Medical Sciences, Uppsala University, Entrance 40 Uppsala University Hospital, 751 85 Uppsala, Sweden

Tel.: +46 738 681133

jan.eriksson@medsci.uu.se

ESM Methods

Inclusion criteria

1. Provision of informed consent prior to any study-specific procedures.
2. Men or women aged ≥40 years and ≤75 years with suitable veins for cannulation or repeated venipuncture.
3. Liver fat content as assessed by MRI >5.5%.
4. Type 2 diabetes diagnosed since ≥6 months in accordance with World Health Organization criteria. Diagnosis of type 2 diabetes could have been based on the following:
   - prior documentation in medical records of type 2 diabetes and/or
   - treatment with anti-hyperglycemic medications and/or diet and/or
   - random plasma glucose ≥11.1 mmol/l or fasting ≥7.0 mmol/l or HbA1c ≥48 mmol/mol (6.5%).
5. Antidiabetic therapy: stable (i.e. >1 months) metformin and/or sulfonylurea or nonpharmacological treatment.
6. For patients without concomitant sulfonylurea: HbA_{1c} ≥48 and ≤80 mmol/mol (≥6.5% and ≤9.5%) at Visit 1. For patients with concomitant sulfonylurea: HbA_{1c} ≥53 and ≤80 mmol/mol (≥7.0% and ≤9.5%) at Visit 1.
7. BMI ≥25 kg/m^2 and ≤40 kg/m^2.

Exclusion criteria

1. Involvement in the planning and/or conduct of the study.
2. Participation in another clinical study with an investigational product during the last 28 days.

3. Any condition when MRI is contraindicated such as, but not limited to, having a pacemaker or claustrophobia.
4. History of or presence of (as found at Visit 1) any clinically significant disease or disorder which, in the opinion of the investigator, may either put the patient at risk because of participation in the study, or influence the results or the patient's ability to participate in the study.
5. Diagnosis or signs of type 1 diabetes (e.g. history of positive islet antibodies).
6. Creatinine clearance <60 ml/min at screening (Cockcroft-Gault formula).
7. Severe hepatic injury and/or significant abnormal liver function defined as aspartate aminotransferase >3× upper limit of normal (ULN) and/or alanine aminotransferase >3× ULN.
8. Total bilirubin >2.0 mg/dl (34.2 μmol/l).
9. Intolerance to omega-3 fatty acids, ethyl esters or fish.
10. Intolerance or allergy to dapagliflozin or any other sodium–glucose co-transporter 2 inhibitor (SGLT2i) or any other substance in the tablets.
11. Use of dapagliflozin or any other SGLT2i within the last 4 weeks prior to Visit 1.
12. Use of insulin or glucagon-like peptide-1 therapy or oral antidiabetic drugs other than metformin or sulfonylurea within the last 4 weeks prior to Visit 1.
13. Use of fish oil, other eicosapentaenoic acid (EPA)- or docosahexaenoic acid (DHA)-containing supplements, or EPA- and/or DHA-fortified foods within 4 weeks from Visit 1, or during the study.
14. Ongoing weight-loss diet (hypocaloric diet) or use of weight-loss agents, unless the diet or treatment has been stopped at least 3 months before screening and that the patient has had a stable body weight (+/– 3 kg) during the 3 months before screening.

15. Use of flax seed, perilla seed, hemp, spirulina or blackcurrant oils within 1 month from study start and during the study until study end.
16. Any clinically significant abnormalities in clinical chemistry, haematology or urinalysis results as judged by the investigator. This includes signs of liver disease other than non-alcoholic fatty liver disease that motivated further investigations or treatment based on clinical judgment.
17. Recent history (past 12 months) of drug abuse or alcohol abuse. Alcohol abuse is defined as >14 drinks per week (1 drink = 35 cl beer, 14 cl wine or 4 cl hard liquor) or as judged by the investigator.
18. Women who are pregnant, lactating or planning to become pregnant during the study period, or women of childbearing potential who are not using acceptable contraceptive methods. A woman is considered of childbearing potential if she is not surgically sterile or is less than 1 year since last menstrual period. Acceptable contraceptive methods were: combined (oestrogen- and progesterone-containing) hormonal contraception associated with inhibition of ovulation (oral, intravaginal, transdermal), progesterone-only hormonal contraception associated with inhibition of ovulation (oral, injectable, implantable), intrauterine device, intrauterine hormone-releasing system, bilateral tubal occlusion and vasectomized.
19. Any other condition the investigator believed would interfere with the patient's ability to provide informed consent, comply with study instructions, or which might confound the interpretation of the study results or put the patient at undue risk.
20. Plasma donation within 1 month of screening or any blood donation/blood loss >500 ml during the 3 months prior to Visit 1 or during the study.

Intervention

Omega-3 carboxylic acid (OM-3CA) capsules Epanova® (OM-3CA) capsules are 1000 mg soft gelatine capsules for oral administration. Each capsule contains a complex mixture of free omega-3 fatty acids and not less than 850 mg of polyunsaturated fatty acids. The predominant omega-3 fatty acids are eicosapentaenoic acid (500–600 mg/g), docosahexaenoic acid (150–250 mg/g) and docosapentaenoic acid (10–80 mg/g). The total amount of n-6 fatty acids is no more than 100 mg/g, and monounsaturated fatty acids no more than 50 mg/g (Epanova® Investigator's Brochure 2013).

Blood analyses Fasting blood samples were taken at baseline and at the end of treatment in the morning before the intake of the investigational products. Plasma glucose levels were analysed using a hexokinase enzymatic method; GLUC3 (Glucose HK Gen. 3) reagent kit (Roche Diagnostics, Indianapolis, IN, USA). Non-esterified fatty acids (NEFA) were analysed using an enzymatic colorimetric assay; NEFA HR (2) test kit (WAKO Chemicals, Richmond, VA, USA). Both glucose and NEFA analyses were performed using the Roche Modular and Cobas Analyzer. Serum levels of total cholesterol and triglycerides were measured using the Cholesterol Gen 2 (CHOL2) reagent and the triglyceride reagent, respectively, from Roche Diagnostics. High-density-lipoprotein (HDL) and low-density-lipoprotein (LDL) cholesterol were measured using direct HDL and LDL cholesterol methods, HDLC3, third-generation reagents and LDL-C plus, second-generation assay (Roche Diagnostics). Levels of cholesterol and triglycerides were measured on the Roche Modular and Cobas Analyzers. Beta-hydroxybutyrate plasma levels were analysed with an enzymatic colorimeteric assay (LiquiColor, Stanbio Laboratory, Boerne, TX, USA). Uric acid was measured using an enzymatic colorimetric assay (ABX Pentra Uric acid CP, Horiba ABX, Montpellier, France). Measurement of HbA_{1c} levels utilized the principles of ion-exchange high-performance liquid chromatography. All Variant II and Variant II Turbo

Hemoglobin A1c reagents were manufactured by Bio-Rad (Hercules, CA, USA). Plasma insulin levels were measured using Access Ultrasensitive Insulin assay, a simultaneous one-step immunoenzymatic (sandwich) assay (Beckman Coulter Inc., Brea, CA, USA). Serum C-peptide levels were analysed by a two-site sandwich immunoassay methodology, ADVIA Centaur C-Peptide ReadyPack (Siemens Healthcare Diagnostics, Tarrytown, NY, USA). Apolipoprotein C-III (ApoCIII) was detected using an immunoturbidimetric methodology from Kamiya Biomedical Company (Seattle, WA, USA). Levels of ApoCIII were measured on the Roche Modular and Cobas Analyzers. The C-reactive protein high-sensitive assay based on immunonephelometry (CRP N HS reagent, Siemens Healthcare Diagnostics, Deerfield, IL, USA) was used to analyse CRP. The Cytokeratin (CK)-18 fragments were measured using the M30-Apoptosense® ELISA and the M65® ELISA solid-phase sandwich enzyme immunoassays (PEVIVA AB, Bromma, Sweden). Adiponectin was quantified by sandwich ELISAs from R&D Systems (Minneapolis, MN, USA). Fibroblast growth factor 21 (FGF21), Leptin, TNF-alpha and osteopontin were quantified using immunoassays from R&D Systems.

Glucose tolerance and insulin sensitivity indices Homeostatic model assessment of insulin resistance (HOMA-IR) was calculated using the following formula: fasting insulin (mU/l) × fasting glucose (mmol/l) / 22.5. An OGTT was performed at baseline (Visit 2) and at the end of the study (Visit 5). A standardized oral glucose load of 75 g was given at time “0”. Plasma samples for measurement of glucose, NEFA and insulin were taken at –15, 0, 30, 60 and 120 minutes. Oral glucose tolerance is indicated by the glucose levels at 120 minutes. The method described by Belfiore et al [2] was used to estimate adipose tissue insulin sensitivity index, ISI(ffa), using the formula ISI(ffa) = 2 / [(INSp × FFAp) + 1]. INSp and FFAp were calculated from the AUC 0–60 minutes and 60–120 minutes for respective measurement.

Fatty acid composition of phospholipids and cholesterol esters and total plasma levels of DHA and EPA The fatty acid composition of phospholipids and cholesterol esters was analysed by first extracting plasma lipids with chloroform and then phospholipids (PL), and cholesterol esters (CE) were separated from other lipids by thin-layer chromatography and trans-methylated with methanol and sulfuric acid, as previously described [3]. The percentage composition of methylated fatty acids was determined by gas chromatography (GC) with flame ionization detection. GC used for the analysis consisted of a 30 m capillary column coated with Thermo TR-FAME (Thermo Electron Corporation, Waltham, MA, USA) and Agilent Technologies (Santa Clara, CA, USA) system (GC 6890N, Autosampler 7683, and Agilent ChemStation). The temperature used was between 150°C and 260°C. The fatty acids were identified by comparing the retention time of each peak with the standard of methyl ester (Nu Check Prep, Elysian, MN, USA) [3].

Desaturase activities were estimated by calculating fatty acid product to precursor ratios in plasma CE: stearoyl-CoA desaturase-1 (SCD-1) as palmitoleic acid (16:1n-7) to palmitic acid (16:0) ratio and oleic acid (18:1n-9) to stearic acid (18:0) ratio, respectively [3]. Delta-5 desaturase and delta-6 desaturase activities were calculated as arachidonic acid to di-homo gamma linolenic acid ratio (20:4n-6/20:3n-6) and gamma-linolenic acid to linoleic acid ratio 18:3n-6/18:2n-6), respectively.

Total (esterified and free plasma levels) EPA and DHA concentrations in plasma were measured by Covance Laboratories Inc. (Madison, WI, USA), on behalf of AstraZeneca. The method used was liquid chromatography with tandem mass spectrometric detection (LC-MS/MS), which was validated over the range 1.00–250 µg/ml, and used appropriate stable label internal standards. EPA and DHA were extracted from plasma after digestion using liquid–liquid extraction, evaporated under nitrogen and subsequently reconstituted and

analysed by LC-MS/MS. The assay had a within-assay and between-assay coefficient of variation of less than or equal to 15%.

***PNPLA3* genotyping** The single nucleotide polymorphism in the *PNPLA3* gene rs738409 was investigated in the 80 participants (*n* = 20 in each group) who gave informed consent for genetic testing. DNA extraction from whole EDTA-treated blood and genotyping using quantitative real-time PCR were performed by Tepnel Pharmaceutical Services (Hologic Ltd, Livingston, UK).

MRI MRI was used to quantify liver lipids using a proton density fat fraction technique utilizing a spoiled three-dimensional gradient, six-echo gradient echo in the axial plane covering the liver in a single breath-hold. The water-fat image reconstruction was performed including T2* and a multi-peak lipid spectrum in the signal model [1]. The liver was segmented manually by a trained operator from the axial slices using the software ImageJ (https://imagej.nih.gov/ij/). The border of the liver was avoided to reduce partial volume effects. The liver fat content was determined by the median of the fat fraction values inside the delineated total liver volume. Liver volume was assessed using a dedicated T1-weighted gradient echo, single echo, single breath-hold scan with high resolution and spectral fat suppression. The full liver was segmented by a trained operator using the semi-automated segmentation software Smartpaint (http://www.cb.uu.se/~filip/SmartPaint/). The coefficient of variation for repeated examinations and analyses of liver PDFF was 5.3% as determined by test-retest scanning and analysis of 10 healthy volunteers.

For determination of abdominal adipose tissue volumes, a 21-slice, 8 mm slice thickness, 3-echo gradient echo axial scan, positioned at the L4/L5, was performed in a single breath-hold. Water and fat images were reconstructed, and visceral adipose tissue (VAT) and subcutaneous adipose tissue (SAT) volumes were extracted using an automated method. Manual inspection

was performed, and results were corrected when needed by an experienced operator. The coefficient of variation for repeated examinations and analyses of SAT and VAT was 0.7% and 2.3%, respectively, as determined by test-retest scanning and analysis of 10 healthy volunteers.

Oxidative stress biomarkers in plasma and urine

Chemicals and reagents

Ammonium acetate, 1,3-cyclohexandione (CHD), butylated hydroxytoluene, acetic acid and methanol (LC-MS grade) were purchased from Merck (Darmstadt, Germany). Formic acid (LC-MS grade) and isopropanol acetonitrile (LC-MS grade) were purchased from Fischer Scientific (Loughborough, UK). An isotopic labelled internal standard mixture of acylcarnitines (NSK-B Acylcarnitine Reference Standards) was purchased from Cambridge Isotope Laboratories, Inc. (MA, USA). Standards of L-acetylcarnitine, L-butyrylcarnitine, L-octanoylcarnitine and L-palmitoylcarnitine were all purchased from Larodan AB (Solna, Sweden). Standards of 4-hydroxy hexenal (4-HHE) and 4-hydroxy nonenal (4-HNE), as well as the corresponding isotopic labelled internal standards (4-hydroxy hexenal-D_3 [4-HHE-D_3] and 4-hydroxy Nonenal-d_3 [4-HNE-D_3]), were all purchased from Cayman Chemical (Ann Arbor, MI, USA). Standards of 2,3-dinor-8-*iso*-PGF2-alpha and 8-*iso*-PGF2-alpha, as well as the corresponding isotopic labelled internal standard 8-*iso*-PGF2-alpha-D_4, were all purchased from Cayman Chemical.

Acylcarnitine analysis

We used a simplified sample pretreatment protocol described by Peng *et al.* [4] where we injected the supernatant of the protein-precipitated plasma sample directly onto the LC-MS system without a drying step. Briefly, plasma samples (50 µl) were aliquoted in 1.2 ml glass vials (Glaswarenfabrik Karl Hecht GmbH & Co KG, Rhön, Germany) and placed in a 96-vial

aluminium rack. Then, 50 µl of internal standard solution (d_9-carnitine [10 mM], d_3-butyrylcarnitine [1 mM], d_3-octanoylcarnitine [1 mM] and d_3-palmitoylcarnitine [1 mM]) was added, followed by 300 µl of acetonitrile/methanol solution (3 volumes/1 volume) using the Agilent Bravo Automated Liquid Handling Platform (Agilent Technologies). After protein precipitation, the samples were vortex-mixed for 30 s and then centrifuged for 10 minutes (4°C) at 4000*g* (Eppendorf 5810R, Germany). The supernatants were transferred to new 1.2 ml glass vials using the Bravo robot and capped using a silicone cap mat to prevent evaporation.

LC-MS analysis was performed using a system comprising an ultra-high-performance liquid chromatography (UHPLC) system (1290 Infinity binary pump, 1290 Infinity autosampler with thermostat and 1290 Infinity thermostated column compartment, all Agilent Technologies) coupled to a 6460 triple quadrupole mass spectrometer (Agilent Technologies). Chromatography was performed on a Poroshell 120 HILIC (100 mm × 2.1 mm i.d., 2.7 µm particle size, 120 Å pore size, Agilent Technologies) at a column temperature of 25°C. A sample volume of 10 µl was injected onto the column at a flow rate of 0.3 ml/min. Eluant A consisted of acetonitrile:water (95:5), and eluent B consisted of 10 mM ammonium acetate with 0.2% formic acid. Gradient elution was performed with the following programme: 15% B to 26% B in 4.5 minutes, wash 50% B during 1 minute followed by re-equilibration at 15% B for 3 minutes. The total cycle time of the run was 9 minutes.

The analytes were measured using multiple reaction monitoring (MRM) operated in positive ion mode, and the following transitions were monitored: L-acetylcarnitine (*m*/*z* 204 →85, collision energy 15 eV), d_3-acetylcarnitine (*m*/*z* 207 → 85, collision energy 15 eV), L-butyrylcarnitine (*m*/*z* 232 → 85, collision energy 17 eV), d_3-buturylcarnitine (*m*/*z* 235 → 85, collision energy 17 eV), L-octanoylcarnitine (*m*/*z* 288 → 85, collision energy 21 eV), d_3-octanoylcarnitine (*m*/*z* 291 → 85, collision energy 21 eV), L-palmitoylcarnitine (*m*/*z* 400 →

85, collision energy 25 eV) and d_3-palmitoylcarnitine (*m/z* 403 → 85, collision energy 25 eV). The instrument settings of the 6460 triple quadrupole mass spectrometer were as follows: drying gas temperature, 325°C; drying gas flow, 9 l/min; nebulizer pressure, 35 psi; capillary voltage, 4000 V, sheath gas temperature 350°C; sheath gas flow, 11 l/min, fragmentor voltage, 110 V; cell accelerator voltage, 4 V; and dwell time, 35 ms. Quadrupoles were working at unit resolution.

Calibration samples were made by spiking a matrix of water/methanol (1:1) with known quantities of acylcarnitine standards and were subjected to the same treatment as the samples described in the sample preparation section above. A six-point calibration curve was obtained by plotting the peak area ratios of corresponding analyte to the internal standard against their theoretical concentrations. The MassHunter Quantitative Analysis Software (version B.06.00, Agilent Technologies) was used for constructing the linear regression analysis with 1/x weighting and for the determination of analyte concentration in the samples.

Aldehyde analysis

The volatility and intrinsically low response of aldehydes in electrospray ionization present analytical challenges; these can be circumvented by chemical derivatization that enables LC-MS/MS measurements to be made with high sensitivity. The derivatization method was based on the protocol described by Matsouka *et al.* [5] in which aldehydes are condensed with CHD and ammonium ions to form water-soluble adducts. Briefly, both the calibration standards and the plasma samples (200 µl) were pipetted into 2 ml microcentrifuge tubes (Eppendorf, Hamburg, Germany) and were subjected to the addition of 125 µl of CHD solution, 125 µl of acetonitrile and 20 µl of internal standard. The mixtures were put into a Thermomixer comfort (Eppendorf GmbH) and heated to 60°C for 60 minutes at a shaking frequency of 750 rpm. After the reaction, the tubes were placed on ice for 1 minute. Next, 600 µl of acetonitrile was added to each tube in order to precipitate the proteins. The tubes were subjected to

centrifugation (12,500 rpm, 8°C) for 10 minutes, and the supernatants were subjected to solid-phase extraction (SPE) using a polymeric reversed-phase approach in a 96-well plate format (Strata-X 33 µm; 60 mg/well, Phenomenex, Torrance, CA, USA). Briefly, the SPE well plate was activated with 2 ml of methanol and reconditioned with 1 ml of 75% aqueous acetonitrile before use. The calibration standards and the samples were loaded onto the SPE plate, and the flow-through fractions were collected into glass vials. The fractions were dried under gentle nitrogen gas flow at –35°C (Techne Sample Concentrator, Staffordshire, UK) and finally reconstituted in 400 µl of 20% acetonitrile.

The analysis was performed on an LC-MS system comprising a UHPLC system (1290 Infinity binary pump, 1290 Infinity autosampler with thermostat and 1290 Infinity thermostated column compartment, all Agilent Technologies) coupled to a 6490 triple quadrupole mass spectrometer (Agilent Technologies). Chromatography was performed on an Eclipse RRHD column (100 mm × 2.1 mm i.d., C18, 1.8 µm particle size, 300 Å pore size; Agilent Technologies) at a column temperature of 45°C. A sample volume of 20 µL was injected onto the column at a flow rate of 0.35 ml/min. Eluant A consisted of 0.1% formic acid in water, and eluent B consisted of acetonitrile with 0.1% formic acid. Gradient elution was performed with the following programme: 12% B to 55% B in 5.5 minutes, wash 95% B during 1.5 minutes followed by re-equilibration at 12% B for 3.5 minutes. The total cycle time of the run was 10.5 minutes.

The analytes were measured using MRM operated in positive ion mode, and the following transitions were monitored: 4-HHE (*m*/*z* 284.1 → 266.1, collision energy 21 eV), 4-HHE-D_3 (*m*/*z* 287.1 → 269.1, collision energy 15 eV), 4-HNE (*m*/*z* 326.1 → 308.1, collision energy 21 eV) and 4-HNE-D_3 (*m*/*z* 329.1 → 311.1, collision energy 21 eV). The instrument settings for the 6490 triple quadrupole mass spectrometer were as follows: drying gas temperature, 150°C; drying gas flow, 15 l/min; nebulizer pressure, 30 psi; capillary voltage, 3500 V; sheath

gas temperature, 250°C; sheath gas flow, 11 l/min; fragmentor voltage, 380 V; dwell time, 35 ms; nozzle voltage, 300 V; cell accelerator voltage, 5 V; high-pressure RF, 200 V; and low-pressure RF, 110 V. Quadrupoles were working at unit resolution.

Calibration samples were made by spiking a matrix of CHD solution with known quantities of 4-HHE/4-HNE standards and were subjected to the same treatment as the samples described in the sample preparation section above. A five-point calibration curve was obtained by plotting the peak area ratios of corresponding analyte to the internal standard against their theoretical concentrations. The MassHunter Quantitative Analysis Software (version B.06.00, Agilent Technologies) was used for constructing the linear regression analysis with 1/x weighting and for the determination of analyte concentration in the samples.

Non-esterified F2 isoprostane analysis

Urine samples (250 µl) were centrifuged at 2500*g* for 5 minutes at 4°C and aliquoted in 2 ml polypropylene tubes. Twenty-five microlitres of internal standard solution (8-*iso*-PGF2-alpha-D_4 [100 mg/ml in methanol]) was added, and the samples were vortex-mixed for 10 s. The samples were transferred to an SPE 96-well plate (Strata-X-AW 33 µm [30 mg/well, Phenomenex]).

The SPE well plate was conditioned with 1.2 ml of 2% formic acid in methanol followed by equilibration with 1.2 ml of water. The samples were loaded onto the SPE plate and washed with 1.2 ml of water followed by 1.2 ml of 20% methanol in water and then 100% acetonitrile. The SPE columns were dried for 30 s, and analytes were eluted with 1.2 ml of methanol. The eluted fractions were collected into glass vials. The fractions were dried under gentle nitrogen gas flow at –35°C (Techne Sample Concentrator) and finally reconstituted in 100 µl of 25% methanol in water.

The analysis was performed on an LC-MS system comprising a UHPLC system (1290 Infinity binary pump, 1290 Infinity autosampler with thermostat and 1290 Infinity thermostated column compartment; all Agilent Technologies) coupled to a 6490 triple quadrupole mass spectrometer (Agilent Technologies). Chromatography was performed on an Acuity UPLC HSS T3 (100 mm × 2.1 mm i.d., 1.8 µm particle size (Waters, Milford, MA, USA) at a column temperature of 40°C. A sample volume of 10 µL was injected onto the column at a flow rate of 0.3 ml/min. Eluent A consisted of acetonitrile:water (25:75) with 0.1% formic acid, and eluent B consisted of acetonitrile:methanol (90:10). Gradient elution was performed with the following programme: 0% B to 40% B in 10 minutes, wash 100% B for 4 minutes and then re-equilibrate at 0% B for 2 minutes. The total cycle time of the run was 16 minutes.

The analytes were measured using MRM operated in negative ion mode, and the following transitions were monitored: 2,3-dinor-8-*iso*-PGF2-alpha (*m/z* 325.2 →237.1, collision energy 10 eV), 8-*iso*-PGF2-alpha (*m/z* 353.0 →193.0, collision energy 20 eV) and 8-*iso*-PGF2-alpha-D_4 (*m/z* 357.0 →197.0, collision energy 20 eV). The instrument settings of the 6490 triple quadrupole mass spectrometer were as follows: drying gas temperature, 210°C; drying gas flow, 16.5 l/min; nebulizer pressure, 40 psi; capillary voltage, 4500 V, sheath gas temperature 375°C; sheath gas flow, 12 l/min; fragmentor voltage, 110 V; cell accelerator voltage, 5 V; and dwell time, 50 ms. Quadrupoles were working at unit resolution.

Normalization to creatinine levels

An aliquot of 100 µl of a urine sample (centrifuged at 2500*g* or 5 minutes at 4°C) was sent for analysis of creatinine. Urine creatinine was measured using an enzymatic colorimetric method (Kit cat no: A11A01933, Horiba ABX, France). The assay was performed in an ABX Pentra 400 instrument (Horiba ABX). The concentrations of 2,3-dinor-8-*iso*-PGF2-alpha and 8-*iso*-

PGF2-alpha were normalized to creatinine and expressed as nanograms per milligram creatinine. Calibration samples were made by spiking methanol with known quantities of 2,3-dinor-8-*iso*-PGF2-alpha and 8-*iso*-PGF2-alpha standards and internal standard (8-*iso*-PGF2-alpha-D_4). The calibration samples were dried and reconstituted in 100 µl of 25% methanol in water.

A nine-point calibration curve was obtained by plotting the peak area ratios of corresponding analyte to the internal standard against their theoretical concentrations. The MassHunter Quantitative Analysis Software (version B.06.00; Agilent Technologies) was used for constructing the linear regression analysis with 1/x weighting and for the determination of analyte concentration in the samples. 8-*Iso*-PGF2-alpha and its isomer 8-*iso*-15(*R*)-PGF2-alpha are quantified together and reported as the amount of 8-*iso*-PGF2-alpha. The concentrations in the urine samples were adjusted for the 10-fold concentration during sample preparation before normalization to creatinine.

ESM References

1. Berglund J, Kullberg J (2012) Three-dimensional water/fat separation and T2* estimation based on whole-image optimization – application in breathhold liver imaging at 1.5 T. Magn Reson Med 67:1684–1693

2. Belfiore F, Iannello S, Volpicelli G (1998) Insulin sensitivity indices calculated from basal and OGTT-induced insulin, glucose, and FFA levels. Mol Genet Metab 63:134–141

3. Rosqvist F, Bjermo H, Kullberg J et al. (2017) Fatty acid composition in serum cholesterol esters and phospholipids is linked to visceral and subcutaneous adipose tissue content in elderly individuals: a cross-sectional study. Lipids Health Dis 16:68

4. Peng M, Liu L, Jiang M et al. (2013) Measurement of free carnitine and acylcarnitines in plasma by HILIC-ESI-MS/MS without derivatization. J Chromatogr B Analyt Technol Biomed Life Sci 932:12–18

5. Matsuoka M, Imado N, Maki T, Banno K, Sato T (1996) Determination of free aliphatic aldehydes in plasma by high-performance liquid chromatography of the 1,3-cyclohexanedione derivatives. Chromatographia 43:501–506

ESM tables

ESM Table 1 Total plasma levels of DHA and EPA

	**Placebo (*n* = 20)**	**OM-3CA (*n* = 13)**	**Dapagliflozin (*n* = 18)**	**OM-3CA + dapagliflozin (*n* = 19)**
**P-DHA, µg/ml**				
B	81.8 (31.1)	84.8 (25.9)	77.0 (23.7)	78.6 (21.5)
C	–1.1 (25.1)	35.4 (25.4)	–2.9 (17.6)	26.5 (31.0)
GMR	0.99 (0.87, 1.13)	1.47 (1.22, 1.78)*	0.95 (0.87, 1.05)	1.34 (1.17, 1.53)*
**P-EPA, µg/ml**				
B	46.5 (18.8)	48.7 (15.5)	41.8 (16.5)	41.3 (12.4)
C	0.2 (24.0)	107.9 (50.3)	0.05 (16.4)	88.1 (47.2)
GMR	0.99 (0.82, 1.20)	3.29 (2.48, 4.36)*	0.96 (0.80, 1.15)	3.09 (2.55, 3.75)*

Data are reported as: B, mean baseline levels (SD); C, mean change from baseline (SD); GMR, descriptive geometric mean ratio (95% CIs)
*$p < 0.05$ vs placebo, mixed model analysis
DHA, docosahexaenoic acid; EPA, eicosapentaenoic acid; OM-3CA, omega-3 carboxylic acids

ESM Table 2 Treatment effects on plasma cholesterol ester fatty acid composition

	Placebo (*n* = 20)	OM-3CA (*n* = 16)	Dapagliflozin (*n* = 19)	OM-3CA + dapagliflozin (*n* = 20)
**14:0**				
B	0.82 (0.24)	0.89 (0.20)	0.76 (0.14)	0.75 (0.17)
C	0.021 (0.15)	0.001 (0.16)	0.011 (0.158)	0.040 (0.228)
GMR	1.04 (0.96, 1.12)	1.00 (0.91, 1.10)	0.99 (0.90, 1.10)	1.04 (0.92, 1.17)
**15:0**				
B	0.25 (0.04)	0.25 (0.05)	0.24 (0.05)	0.24 (0.04)
C	0.02 (0.05)	0.02 (0.05)	–0.001 (0.05)	0.02 (0.04)
GMR	1.09 (1.00, 1.20)	1.07 (0.96, 1.19)	0.99 (0.89, 1.09)*	1.08 (1.00, 1.17)
**16:0**				
B	11.6 (0.80)	11.6 (0.62)	11.7 (0.87)	11.5 (0.50)
C	0.07 (0.67)	0.83 (0.77)	–0.32 (0.82)	0.71 (0.80)
GMR	1.01 (0.98, 1.03)	1.07 (1.03, 1.11)*	0.97 (0.94, 1.01)	1.06 (1.03, 1.09)*
**16:1n-7**				
B	3.5 (1.4)	4.0 (1.1)	3.6 (1.5)	3.2 (0.90)
C	–0.16 (0.61)	–0.59 (0.76)	–0.37 (0.95)	–0.69 (0.57)
GMR	0.96 (0.88, 1.05)	0.84 (0.76, 0.93)	0.90 (0.80, 1.01)	0.78 (0.72, 0.85)*
**18:0**				
B	0.81 (0.18)	0.83 (0.15)	0.84 (0.24)	0.76 (0.16)
C	–0.03 (0.17)	0.03 (0.23)	–0.09 (0.24)	0.07 (0.22)
GMR	0.96 (0.88, 1.05)	1.03 (0.90, 1.18)	0.92 (0.82, 1.03)	1.10 (0.98, 1.23)
**18:1n-9**				
B	23.8 (1.6)	23.6 (1.8)	24.4 (1.7)	23.4 (2.2)
C	0.15 (0.90)	–0.35 (1.4)	0.05 (1.4)	–0.80 (2.0)
GMR	1.00 (0.99, 1.02)	0.98 (0.95, 1.01)	1.00 (0.97, 1.03)	0.97 (0.93, 1.01)*
**18:2n-6**				
B	45.9 (4.0)	45.7 (3.2)	45.8 (3.2)	45.9 (3.5)
C	–0.14 (3.2)	–4.37 (3.8)	0.86 (2.2)	–2.77 (3.8)
GMR	1.00 (0.96, 1.03)	0.90 (0.86, 0.95)*	1.02 (0.99, 1.04)	0.94 (0.90, 0.98)*
**18:3n-6**				
B	0.99 (0.37)	1.13 (0.33)	0.97 (0.39)	1.06 (0.37)
C	–0.06 (0.22)	–0.46 (0.26)	–0.03 (0.36)	–0.39 (0.26)
GMR	0.94 (0.85, 1.03)	0.60 (0.53, 0.67)*	0.97 (0.81, 1.16)	0.61 (0.52, 0.71)*
**18:3n-3**				
B	0.81 (0.20)	0.87 (0.21)	0.84 (0.20)	0.79 (0.25)
C	0.002 (0.15)	–0.04 (0.21)	0.04 (0.19)	–0.02 (0.30)

	Placebo (*n* = 20)	OM-3CA (*n* = 16)	Dapagliflozin (*n* = 19)	OM-3CA + dapagliflozin (*n* = 20)
GMR	0.99 (0.90, 1.08)	0.96 (0.83, 1.12)	1.05 (0.95, 1.17)	1.00 (0.81, 1.23)
**20:3n-6**				
B	0.79 (0.12)	0.84 (0.16)	0.85 (0.22)	0.80 (0.09)
C	–0.01 (0.07)	–0.24 (0.16)	–0.09 (0.08)	–0.23 (0.10)
GMR	0.98 (0.93, 1.02)	0.72 (0.64, 0.80)*	0.89 (0.85, 0.93)	0.70 (0.65, 0.76)*
**20:4n-6**				
B	7.6 (1.6)	7.4 (1.4)	7.2 (1.3)	8.6 (1.5)
C	0.02 (0.64)	–0.08 (0.78)	0.04 (0.85)	–0.58 (1.14)
GMR	1.00 (0.96, 1.04)	0.99 (0.94, 1.05)	1.01 (0.96, 1.06)	0.94 (0.88, 1.00)
**20:5n-3 (EPA)**				
B	2.3 (0.8)	2.1 (0.5)	2.0 (0.7)	2.1 (0.5)
C	0.11 (1.17)	4.91 (2.14)	–0.02 (0.56)	4.41 (1.94)
GMR	1.03 (0.85, 1.25)	3.28 (2.62, 4.10)*	0.97 (0.84, 1.13)	2.92 (2.46, 3.46)*
**22:6n-3 (DHA)**				
B	0.84 (0.22)	0.80 (0.19)	0.83 (0.20)	0.89 (0.19)
C	0.02 (0.17)	0.34 (0.20)	–0.08 (0.16)	0.23 (0.20)
GMR	1.03 (0.94, 1.12)	1.43 (1.29, 1.60)*	0.91 (0.82, 1.01)*	1.26 (1.14, 1.39)*

Data are % of total fatty acid reported as: B, mean baseline levels (SD); C, mean change from baseline (SD); GMR, descriptive geometric mean ratio (95% CIs)
*$p < 0.05$ vs placebo, mixed model analysis
DHA, docosahexaenoic acid; EPA, eicosapentaenoic acid; OM-3CA, omega-3 carboxylic acids

ESM Table 3 Treatment effects on plasma phospholipid fatty acid composition

	**Placebo (*n* = 20)**	**OM-3CA (*n* = 16)**	**Dapagliflozin (*n* = 19)**	**OM-3CA + dapagliflozin (*n* = 20)**
**14:0**				
B	0.38 (0.08)	0.41 (0.07)	0.35 (0.06)	0.35 (0.07)
C	0.02 (0.09)	–0.04 (0.06)	0.01 (0.10)	–0.01 (0.08)
GMR	1.05 (0.95, 1.16)	0.91 (0.84, 0.99)	1.02 (0.89, 1.16)	0.95 (0.85, 1.07)*
**15:0**				
B	0.25 (0.08)	0.23 (0.07)	0.22 (0.08)	0.25 (0.10)
C	0.02 (0.09)	–0.01 (0.08)	0.01 (0.08)	–0.003 (0.09)
GMR	1.09 (0.94, 1.26)	0.94 (0.81, 1.09)*	1.06 (0.91, 1.25)	1.02 (0.89, 1.16)
**16:0**				
B	30.0 (1.3)	30.2 (1.2)	30.1 (1.3)	29.8 (1.2)
C	–0.01 (0.85)	–0.19 (0.59)	–0.17 (0.69)	0.19 (0.85)
GMR	1.00 (0.99, 1.01)	0.99 (0.98, 1.00)	0.99 (0.98, 1.00)	1.01 (0.99, 1.02)
**16:1n-7**				
B	0.60 (0.28)	0.67 (0.18)	0.61 (0.29)	0.52 (0.14)
C	–0.05 (0.13)	–0.15 (0.13)	–0.06 (0.22)	–0.10 (0.11)
GMR	0.93 (0.84, 1.03)	0.77 (0.69, 0.87)	0.93 (0.79, 1.08)	0.79 (0.70, 0.89)*
**17:0**				
B	0.40 (0.06)	0.38 (0.05)	0.40 (0.08)	0.41 (0.07)
C	–0.01 (0.04)	0.02 (0.04)	0.01 (0.05)	0.03 (0.04)
GMR	0.99 (0.94, 1.04)	1.05 (1.00, 1.11)	1.04 (0.98, 1.10)	1.07 (1.03, 1.12)*
**18:0**				
B	13.2 (0.8)	13.7 (1.1)	13.5 (1.1)	13.4 (1.2)
C	–0.10 (0.58)	0.17 (0.58)	–0.20 (0.59)	0.07 (0.63)
GMR	0.99 (0.97, 1.01)	1.01 (0.99, 1.04)	0.99 (0.96, 1.01)	1.01 (0.98, 1.03)
**18:1n-9**				
B	12.6 (1.3)	12.5 (1.1)	13.0 (1.1)	12.0 (1.1)
C	–0.13 (0.77)	–1.00 (0.89)	0.20 (1.06)	–0.96 (1.04)
GMR	0.99 (0.96, 1.02)	0.92 (0.88, 0.95)*	1.01 (0.97, 1.05)	0.92 (0.88, 0.96)*
**18:2n-6**				
B	17.5 (2.0)	17.4 (1.8)	17.9 (2.1)	17.2 (1.8)
C	0.25 (1.74)	–2.15 (2.04)	0.39 (1.37)	–1.80 (1.84)
GMR	1.01 (0.96, 1.06)	0.87 (0.81, 0.93)*	1.02 (0.99, 1.06)	0.90 (0.85, 0.94)*
**18:3n-6**				
B	0.11 (0.04)	0.12 (0.03)	0.11 (0.04)	0.11 (0.04)
C	–0.01 (0.03)	–0.05 (0.03)	0.003 (0.04)	–0.04 (0.03)
GMR	0.91 (0.79, 1.04)	0.62 (0.53, 0.73)*	1.00 (0.83, 1.20)	0.63 (0.53, 0.76)*

	Placebo (*n* = 20)	OM-3CA (*n* = 16)	Dapagliflozin (*n* = 19)	OM-3CA + dapagliflozin (*n* = 20)
**18:3n-3**				
B	0.24 (0.06)	0.30 (0.07)	0.27 (0.08)	0.24 (0.09)
C	0.04 (0.09)	–0.05 (0.08)	0.01 (0.11)	–0.03 (0.10)
GMR	1.12 (0.95, 1.33)	0.80 (0.69, 0.94)	1.03 (0.86, 1.23)	0.92 (0.75, 1.14)*
**20:0**				
B	0.73 (0.12)	0.71 (0.09)	0.75 (0.13)	0.71 (0.11)
C	0.000 (0.07)	0.03 (0.05)	–0.01 (0.10)	0.03 (0.06)
GMR	1.00 (0.95, 1.05)	1.03 (0.99, 1.08)	0.98 (0.92, 1.05)	1.05 (1.01, 1.09)
**20:3n-6**				
B	3.2 (0.6)	3.4 (0.6)	3.3 (0.8)	3.1 (0.4)
C	–0.11 (0.34)	–1.14 (0.54)	–0.22 (0.33)	–0.95 (0.52)
GMR	0.96 (0.91, 1.01)	0.66 (0.60, 0.73)*	0.93 (0.88, 0.98)	0.68 (0.62, 0.75)*
**20:4n-6**				
B	9.6 (1.8)	9.4 (1.5)	9.0 (1.4)	10.8 (1.6)
C	–0.11 (0.70)	–0.92 (0.79)	0.16 (0.96)	–1.28 (1.04)
GMR	0.99 (0.96, 1.02)	0.90 (0.86, 0.95)*	1.02 (0.97, 1.07)	0.88 (0.84, 0.92)*
**20:5n-3 (EPA)**				
B	2.2 (0.62)	2.1 (0.48)	1.8 (0.59)	2.0 (0.47)
C	0.17 (1.04)	3.75 (1.67)	0.14 (0.54)	3.60 (1.58)
GMR	1.04 (0.88, 1.23)	2.73 (2.27, 3.29)*	1.06 (0.93, 1.22)	2.68 (2.29, 3.14)*
**22:0**				
B	1.8 (0.35)	1.7 (0.25)	1.8 (0.40)	1.7 (0.31)
C	0.01 (0.18)	0.06 (0.16)	–0.04 (0.19)	–0.003 (0.26)
GMR	1.01 (0.96, 1.05)	1.04 (0.98, 1.09)	0.98 (0.92, 1.03)	1.00 (0.95, 1.06)
**22:5n-3**				
B	1.1 (0.23)	1.1 (0.13)	1.1 (0.16)	1.1 (0.13)
C	–0.02 (0.14)	0.52 (0.31)	–0.03 (0.16)	0.40 (0.16)
GMR	0.99 (0.93, 1.06)	1.46 (1.31, 1.63)*	0.97 (0.91, 1.04)	1.36 (1.29, 1.44)*
**22:6n-3 (DHA)**				
B	4.6 (0.99)	4.4 (1.02)	4.4 (0.99)	4.7 (0.84)
C	0.08 (0.81)	1.13 (0.92)	–0.16 (0.77)	0.84 (0.82)
GMR	1.02 (0.94, 1.11)	1.28 (1.14, 1.43)*	0.97 (0.89, 1.06)	1.19 (1.10, 1.29)*
**24:0**				
B	1.6 (0.30)	1.4 (0.25)	1.5 (0.30)	1.5 (0.24)
C	–0.02 (0.20)	0.07 (0.17)	–0.04 (0.14)	0.03 (0.26)
GMR	0.99 (0.93, 1.04)	1.06 (0.99, 1.12)	0.98 (0.93, 1.02)	1.02 (0.95, 1.10)

Data are reported as: B, mean baseline levels (SD); C, mean change from baseline (SD); GMR, descriptive geometric mean ratio (95% CIs)
*$p < 0.05$ vs placebo, mixed model analysis
DHA, docosahexaenoic acid; EPA, eicosapentaenoic acid; OM-3CA, omega-3 carboxylic acids

ESM Table 4 Estimates of delta-5-, delta-6- and delta-9 desaturase activities from fatty acid composition of plasma phospholipids

	**Placebo (*n* = 20)**	**OM-3CA (*n* = 16)**	**Dapagliflozin (*n* = 19)**	**OM-3CA + dapagliflozin (*n* = 20)**
**20:4n-6/20:3n-6 (delta-5 desaturase)**				
B	3.14 (0.82)	2.88 (0.72)	2.82 (0.69)	3.53 (0.73)
C	0.08 (0.35)	1.05 (0.72)	0.30 (0.62)	1.09 (1.01)
GMR	1.03 (0.98, 1.08)	1.37 (1.23, 1.51)*	1.10 (1.02, 1.18)	1.30 (1.16, 1.44)*
**18:3n-6/18:2n-6 (delta-6 desaturase)**				
B	0.006 (0.003)	0.007 (0.002)	0.006 (0.003)	0.006 (0.002)
C	–0.001 (0.002)	–0.002 (0.002)	0.000 (0.003)	–0.002 (0.002)
GMR	0.90 (0.78, 1.03)	0.71 (0.60, 0.84)	0.97 (0.81, 1.17)	0.70 (0.58, 0.85)*
**16:1n-7/16:0 (delta-9 desaturase)**				
B	0.020 (0.009)	0.022 (0.006)	0.020 (0.009)	0.017 (0.005)
C	–0.002 (0.004)	–0.005 (0.004)	–0.002 (0.007)	–0.004 (0.004)
GMR	0.93 (0.85, 1.03)	0.78 (0.69, 0.87)	0.93 (0.80, 1.09)	0.79 (0.70, 0.88)*
**18:1n-9/18:0 (delta-9 desaturase)**				
B	0.955 (0.148)	0.921 (0.132)	0.966 (0.125)	0.906 (0.112)
C	–0.0004 (0.0789)	–0.090 (0.088)	0.028 (0.102)	–0.078 (0.086)
GMR	1.00 (0.96, 1.04)	0.90 (0.86, 0.95)*	1.03 (0.98, 1.08)	0.91 (0.87, 0.96)*

Data are reported as: B, mean baseline levels (SD); C, mean change from baseline (SD); GMR, descriptive geometric mean ratio (95% CIs)

*$p < 0.05$ vs placebo, mixed model analysis

OM-3CA, omega-3 carboxylic acids

ESM Table 5 Treatment effects on plasma lipoproteins and products of fatty acid oxidation

	**Placebo (*n* = 20)**	**OM-3CA (*n* = 16)**	**Dapagliflozin (*n* = 19)**	**OM-3CA + dapagliflozin (*n* = 19)**
**Total cholesterol, mmol/l**				
B	4.49 (0.91)	4.98 (1.01)	4.87 (1.06)	4.27 (0.70)
C	–0.02 (0.54)	–0.13 (0.48)	0.12 (0.68)	0.05 (0.68)
GMR	0.99 (0.94, 1.05)	0.96 (0.90, 1.04)	1.02 (0.96, 1.09)	1.01 (0.94, 1.08)
**HDL-C, mmol/l**				
B	1.33 (0.384)	1.29 (0.365)	1.29 (0.246)	1.33 (0.264)
C	–0.01 (0.130)	0.01 (0.082)	0.01 (0.124)	0.04 (0.129)
GMR	0.99 (0.94, 1.04)	1.00 (0.96, 1.05)	1.01 (0.96, 1.06)	1.02 (0.97, 1.07)
**LDL-C, mmol/l**				
B	2.54 (0.89)	2.89 (0.89)	2.83 (0.90)	2.30 (0.60)
C	0.04 (0.40)	0.06 (0.45)	0.20 (0.53)	0.15 (0.56)
GMR	1.01 (0.93, 1.11)	1.01 (0.92, 1.11)	1.07 (0.98, 1.16)	1.06 (0.95, 1.18)
**Total triglycerides, mmol/l**				
B	1.91 (0.95)	2.11 (0.92)	2.01 (1.17)	1.90 (0.82)
C	–0.13 (0.515)	–0.18 (0.535)	0.16 (0.457)	–0.29 (0.645)
GMR	0.95 (0.83, 1.08)	0.91 (0.79, 1.05)	1.06 (0.94, 1.19)	0.84 (0.71, 0.99)
**ApoCIII, mg/l**				
B	124 (51)	140 (46)	131 (48)	129 (43)
C	–9 (22)	–10 (26)	17 (32)	1 (34)
GMR	0.93 (0.84, 1.02)	0.95 (0.85, 1.05)	1.14 (1.02, 1.27)*	1.00 (0.88, 1.13)
**β-hydroxybutyrate, µmol/l**				
B	133 (111)	88 (54)	110 (69)	132 (106)
C	27 (119)	–5 (45)	104 (345)	76 (98)
GMR	1.08 (0.79, 1.47)	1.04 (0.80, 1.35)	1.40 (0.99, 1.98)	1.52 (1.16, 1.99)
**Acetylcarnitine, µmol/l**				
B	10.36 (3.64)	9.63 (2.02)	9.14 (2.50)	9.16 (2.09)
C	–0.34 (2.12)	–1.91 (2.44)	1.16 (2.26)	0.78 (2.73)
GMR	0.95 (0.85, 1.06)	0.79 (0.66, 0.95)	1.11 (0.98, 1.27)	1.08 (0.94, 1.25)
**Butyrylcarnitine, µmol/l**				
B	0.31 (0.14)	0.30 (0.09)	0.31 (0.11)	0.32 (0.23)
C	0.01 (0.07)	–0.02 (0.09)	0.08 (0.16)	0.01 (0.11)
GMR	1.02 (0.91, 1.15)	0.92 (0.79, 1.06)	1.24 (1.03, 1.50)*	1.06 (0.89, 1.26)
**Octanoylcarnitine, µmol/l**				
B	0.21 (0.104)	0.19 (0.096)	0.18 (0.093)	0.18 (0.084)

C	0.01 (0.102)	–0.03 (0.099)	0.001 (0.056)	0.03 (0.068)
GMR	0.99 (0.83, 1.18)	0.88 (0.73, 1.07)	1.01 (0.84, 1.22)	1.17 (1.00, 1.39)
**Hexadecanoylcarnitine, µmol/l**				
B	0.12 (0.023)	0.12 (0.025)	0.13 (0.034)	0.12 (0.028)
C	–0.005 (0.026)	–0.017 (0.026)	0.005 (0.032)	0.004 (0.025)
GMR	0.96 (0.86, 1.06)	0.85 (0.74, 0.98)	1.05 (0.93, 1.19)	1.03 (0.93, 1.12)

Data are reported as: B, mean baseline levels (SD); C, mean change from baseline (SD); GMR, descriptive geometric mean ratio (95% CIs)
*$p < 0.05$ vs placebo, mixed model analysis
ApoCIII, apolipoprotein C3; HDL-C, high-density lipoprotein cholesterol; LDL-C, low-density lipoprotein cholesterol; OM-3CA, omega-3 carboxylic acids

ESM Table 6 Treatment effects on hepatocyte damage biomarkers, oxidative stress biomarkers and uric acid

	**Placebo (*n* = 20)**	**OM-3CA (*n* = 14)**	**Dapagliflozin (*n* = 20)**	**OM-3CA + dapagliflozin (*n* = 19)**
**AST, µkat/l**				
B	0.49 (0.22)	0.51 (0.17)	0.52 (0.19)	0.50 (0.17)
C	–0.02 (0.12)	0.08 (0.15)	–0.07 (0.09)	0.02 (0.09)
GMR	0.99 (0.89, 1.11)	1.13 (0.99, 1.28)	0.86 (0.79, 0.94)*	1.03 (0.95, 1.11)
**ALT µkat/l**				
B	0.57 (0.21)	0.64 (0.25)	0.67 (0.25)	0.61 (0.29)
C	–0.003 (0.15)	0.10 (0.28)	–0.14 (0.14)	0.001 (0.22)
GMR	1.00 (0.88, 1.13)	1.09 (0.90, 1.32)	0.78 (0.68, 0.89)*	1.00 (0.88, 1.15)
**Gamma-GT, µkat/l**				
B	0.54 (0.29)	0.90 (0.96)	0.97 (0.72)	0.67 (0.24)
C	0.04 (0.16)	0.04 (0.20)	–0.08 (0.23)	–0.01 (0.23)
GMR	1.17 (0.96, 1.42)	1.00 (0.87, 1.15)	0.89 (0.80, 0.99)*	0.95 (0.80, 1.12)
**CK 18-M30, U/l**				
B	324 (302)	245 (138)	302 (207)	283 (193)
C	48 (95)	131 (217)	–28 (221)	29 (91)
GMR	1.20 (1.03, 1.39)	1.44 (1.04, 1.98)	0.92 (0.73, 1.18)*	1.08 (0.94, 1.23)
**CK 18-M65, U/l**				
B	558 (230)	549 (263)	621 (364)	689 (537)
C	106 (227)	222 (331)	–154 (259)	–110 (570)
GMR	1.14 (1.00, 1.30)	1.44 (1.09, 1.90)	0.79 (0.66, 0.95)*	0.91 (0.69, 1.21)
**Uric acid, µmol/l**				
B	365 (75)	370 (83)	373 (69)	344 (78)
C	–3.0 (38.2)	6.2 (78.1)	–77.0 (52.0)	–47.0 (77.1)
GMR	0.99 (0.94, 1.04)	1.05 (0.89, 1.23)	0.78 (0.72, 0.85)*	0.86 (0.77, 0.96)*
**2-hydroxynonenal, pg/ml**				
B	5.74 (1.14)	5.74 (1.53)	5.51 (1.57)	6.14 (1.13)
C	–0.17 (0.69)	–0.31 (0.79)	–0.08 (0.84)	–0.56 (0.96)
GMR	0.97 (0.91, 1.03)	0.94 (0.87, 1.02)	1.00 (0.92, 1.08)	0.90 (0.83, 0.97)
**2-hydroxyhexenal, pg/ml**				
B	3.76 (1.35)	3.90 (1.23)	3.30 (0.69)	3.52 (0.82)
C	–0.03 (1.15)	2.82 (1.91)	0.11 (0.58)	2.81 (2.40)
GMR	1.00 (0.86, 1.15)	1.73 (1.40, 2.14)	1.02 (0.94, 1.12)	1.73 (1.47, 2.02)*
**U-8-*iso*-PGF2-alpha, ng/mg creatinine**[a]				
B	0.088 (0.068)	0.073 (0.029)	0.073 (0.024)	0.080 (0.029)
C	–0.008 (0.033)	–0.013 (0.021)	0.001 (0.022)	–0.004 (0.022)
GMR	0.96 (0.83, 1.10)	0.83 (0.69, 1.00)	1.02 (0.90, 1.16)	0.95 (0.84, 1.09)
**U-2,3-dinor-8-*iso*-PGF2-alpha, ng/mg creatinine**[a]				
B	1.58 (0.79)	2.18 (1.42)	1.43 (0.70)	1.61 (0.59)
C	0.11 (0.43)	–0.43 (0.92)	0.37 (0.65)	0.05 (0.80)
GMR	1.09 (0.91, 1.30)	0.84 (0.65, 1.08)	1.23 (0.99, 1.54)	1.04 (0.83, 1.31)

Data are reported as: B, mean baseline levels (SD); C, mean change from baseline (SD); GMR, descriptive geometric mean ratio (95% CIs)
[a] Urinary levels, all other analyses are in plasma
*$p < 0.05$ vs placebo, mixed model analysis
ALT, alanine aminotransferase; AST, aspartate transaminase; CK 18-M30, cytokeratin 18-M30; CK 18-M65, cytokeratin 18-M65; gamma-GT, gamma-glutamyl transferase; OM-3CA, omega-3 carboxylic acids

ESM Table 7 Treatment effects on inflammatory biomarkers and hormones

	Placebo (n = 20)	OM-3CA (n = 16)[a]	Dapagliflozin (n = 19)	OM-3CA + dapagliflozin (n = 20)
**C-reactive protein, mg/dl**				
B	1.88 (1.89)	4.20 (3.67)	2.47 (1.96)	3.38 (4.65)
C	0.16 (2.23)	–1.40 (3.44)	–0.23 (1.15)	–1.49 (4.78)
GMR	1.03 (0.69, 1.54)	0.67 (0.37, 1.20)	0.92 (0.73, 1.16)	0.93 (0.59, 1.47)
**Osteopontin, ng/ml**				
B	60.6 (23.3)	71.8 (33.1)	69.2 (46.9)	64.4 (24.4)
C	–9.0 (24.5)	–7.2 (35.3)	–12.4 (42.2)	9.6 (33.6)
GMR	0.82 (0.64, 1.05)	0.84 (0.60, 1.18)	0.88 (0.59, 1.30)	1.14 (0.92, 1.42)*
**FGF21, pg/ml**				
B	339 (248)	515 (269)	378 (184)	392 (219)
C	78 (296)	38 (211)	–7 (234)	–13 (171)
GMR	1.24 (0.99, 1.58)	1.02 (0.82, 1.27)	0.79 (0.61, 1.03)*	1.03 (0.86, 1.22)
**Adiponectin, µg/l**				
B	5591 (3798)	4848 (3021)	4978 (3142)	4882 (3615)
C	–132 (1165)	320 (812)	–298 (1512)	233 (1035)
GMR	1.00 (0.90, 1.11)	1.06 (0.98, 1.14)	0.96 (0.82, 1.13)	1.08 (0.98, 1.20)
**Leptin, µg/l**				
B	16.8 (15.9)	29.7 (22.2)	15.5 (13.3)	19.5 (14.1)
C	0.38 (3.48)	–1.69 (10.48)	–0.45 (4.99)	–1.06 (5.16)
GMR	1.03 (0.92, 1.15)	1.00 (0.85, 1.19)	0.98 (0.83, 1.15)	0.92 (0.80, 1.06)

Data are reported as: B, mean baseline levels (SD); C, mean change from baseline (SD); GMR, descriptive geometric mean ratio (95% CIs)

[a] Analyses of C-reactive protein, adiponectin and leptin, n = 13

*$p < 0.05$ vs placebo, mixed model analysis

FGF21, fibroblast growth factor 21; OM-3CA, omega-3 carboxylic acids

ESM Figures

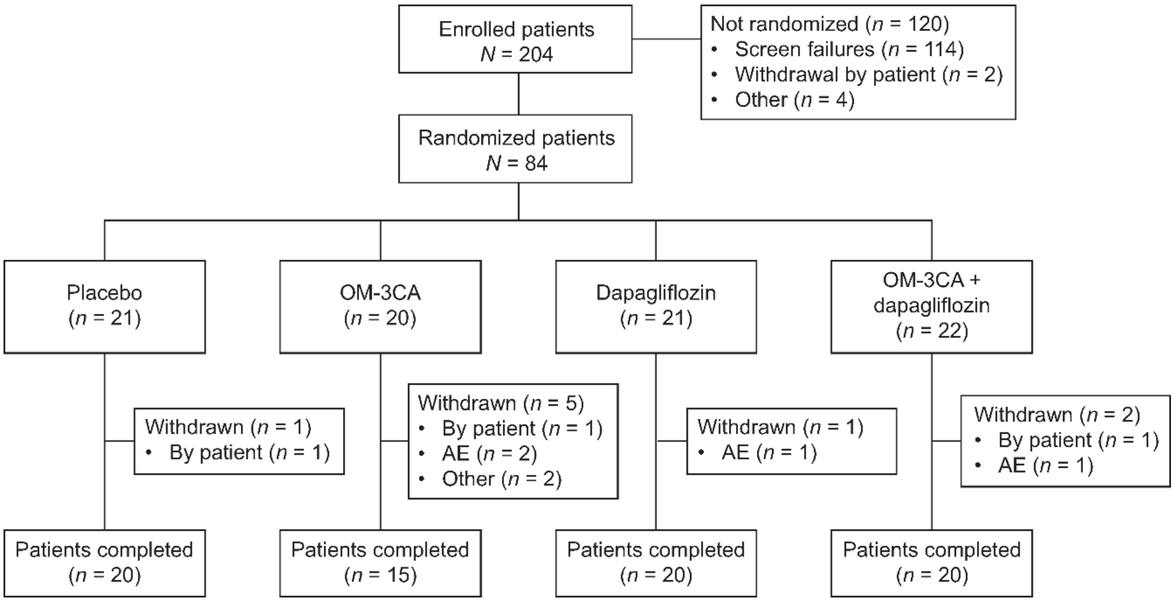

ESM Fig. 1 CONSORT flow chart. AE, adverse event; OM-3CA, omega-3 carboxylic acids

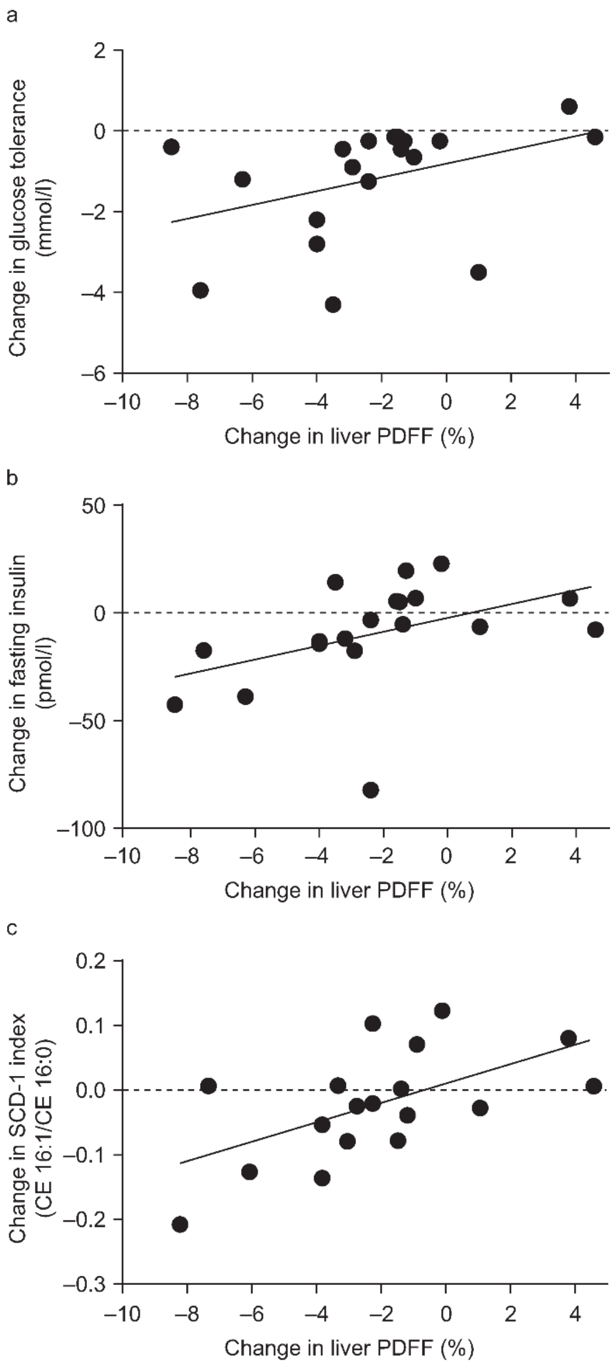

ESM Fig. 2 Correlation plots based on changes in the dapagliflozin treatment group. Change in liver PDFF vs change in glucose tolerance measured as glucose levels at 120 min during OGTT (a), change in liver PDFF vs change in fasting plasma insulin levels (b), and change in liver PDFF vs change in SCD-1 index, measured as 16:1/16:0 in the CE fraction (c). The correlations were analysed using Spearman’s rank correlation test

CE, cholesterol ester; PDFF, proton density fat fraction; SCD-1, stearoyl-CoA desaturase
